# Seasonal changes in the diversity and composition of the litter fauna in native forests and rubber plantations

**DOI:** 10.1038/s41598-018-28603-7

**Published:** 2018-07-06

**Authors:** Kingsly C. Beng, Richard T. Corlett, Kyle W. Tomlinson

**Affiliations:** Center for Integrative Conservation, Xishuangbanna Tropical Botanical Garden, Chinese Academy of Sciences, Menglun, Mengla, Yunnan 666303 China

## Abstract

The litter layer of tropical forests supports a significant fraction of total arthropod diversity and decomposition of this layer is the main pathway by which nutrients are returned to the soil and CO_2_ to the atmosphere. Conversion of tropical forests to agriculture is the main threat to biodiversity and ecosystem services, and understanding effects on the litter layer is important for understanding and mitigating these impacts. We used high through-put DNA sequencing of the mitochondrial cytochrome c oxidase subunit I (COI) gene to assess seasonal changes in the diversity and composition of the litter fauna at five matched pairs of native forests and rubber plantations in tropical SW China every month for a year, and measured the environmental factors expected to drive intra-annual variation. Forests and rubber had very different arthropod assemblages throughout the year, with forests more species-rich than rubber in all months except February. Very high rates of intra-annual turnover in species composition in both forests and rubber were associated with seasonality in environmental variables, with the influence of particular variables differing among taxa. Tropical arthropods are very sensitive to seasonality and sampling at only one time of the year captures only a subset of the total community.

## Introduction

Tropical forests are believed to support more than half of all terrestrial plant and animal species^1^, and are a crucial component of the global carbon cycle, accounting for 25% of the terrestrial carbon pool and one-third of net primary production^2^. Their on-going conversion to agricultural monocultures thus threatens both biodiversity and carbon stocks on a global scale. Within tropical forests, the litter layer supports a significant fraction of total arthropod diversity^1^ and decomposition of this layer is the main pathway by which nutrients are returned to the soil and carbon dioxide to the atmosphere^3^. Litter decomposition is ultimately a microbial process, but invertebrates play key roles by comminuting large particles and facilitating microbial action, as well as by feeding on microbial biomass^4,5^. Understanding the effects of land-use change on the litter layer is thus likely to be crucial for understanding and potentially mitigating the impacts of forest conversion on biodiversity and nutrient cycling.

The litter layer is a challenging system to study because of the high species richness and exceptional phylogenetic diversity of the fauna. Most research has therefore either used litter bags with mesh sizes chosen to exclude invertebrates of different sizes^3^, or has focused on a single taxonomic group^6–8^. Recently, however, DNA metabarcoding has been used to overcome the challenge of high taxonomic diversity and to give a broader view of the diversity and composition of the litter fauna^9–11^. We have previously applied this technique in the fragmented forest landscape of tropical southwest China to compare the litter arthropod fauna in native forests with that in adjacent plantations of tea and rubber, showing a decline in the diversity of most taxonomic groups and large changes in species composition following forest conversion^10^.

The climate of Xishuangbanna is strongly seasonal with a 5–6 month dry season. Litter faunas are known to vary seasonally in other parts of the tropics with similarly strong rainfall seasonality^12,13^. Litter decomposition also varies seasonally in our study area^3^, which may, at least in part, reflect seasonal changes in the litter fauna. Moreover, the temporal turnover in the species composition of the litter fauna means that a significant proportion of the total faunal diversity may be overlooked in studies that ignore seasonality. Previous studies assessing seasonal changes in arthropod diversity have reported contrasting results, mainly due to the limited taxonomic scope of individual assessments. For instance, Grimbacher & Stork^14^ found higher beetle diversity during the pre-wet season, Jacquemin, Roisin & Leponce^15^ found higher litter and soil-dwelling ant richness during the dry season, while Montine *et al*.^16^ did not find any significant difference in ant richness between the dry and rainy seasons. Interestingly, Marin *et al*.^17^ found higher spider richness during the dry season in high-shade coffee and higher spider richness during the rainy season in low-shade coffee.

In Xishuangbanna, forest trees are almost all evergreen, but rubber trees are briefly deciduous, shedding their leaves for a short (2–4 weeks) period in January-February during the dry season, but retaining full foliage throughout the rest of the year. In this study, therefore, we have used the same methods as the previous study^10^ to investigate seasonal changes in the litter fauna at five matched pairs of native forests and rubber plantations by re-sampling at monthly intervals over one year. We address four main questions: (i) Does arthropod species composition, richness and turnover show significant intra-annual variability? (ii) Do these patterns of intra-annual variability differ among arthropod taxa and between forest and rubber? (iii) Which environmental variables are most strongly associated with the temporal patterns of arthropod richness and composition? (iv) How many times per year is it necessary to sample in order to capture temporal variation in species composition in a seasonal tropical climate?

## Results

### Arthropod community composition, richness and turnover

**Figure 1:**
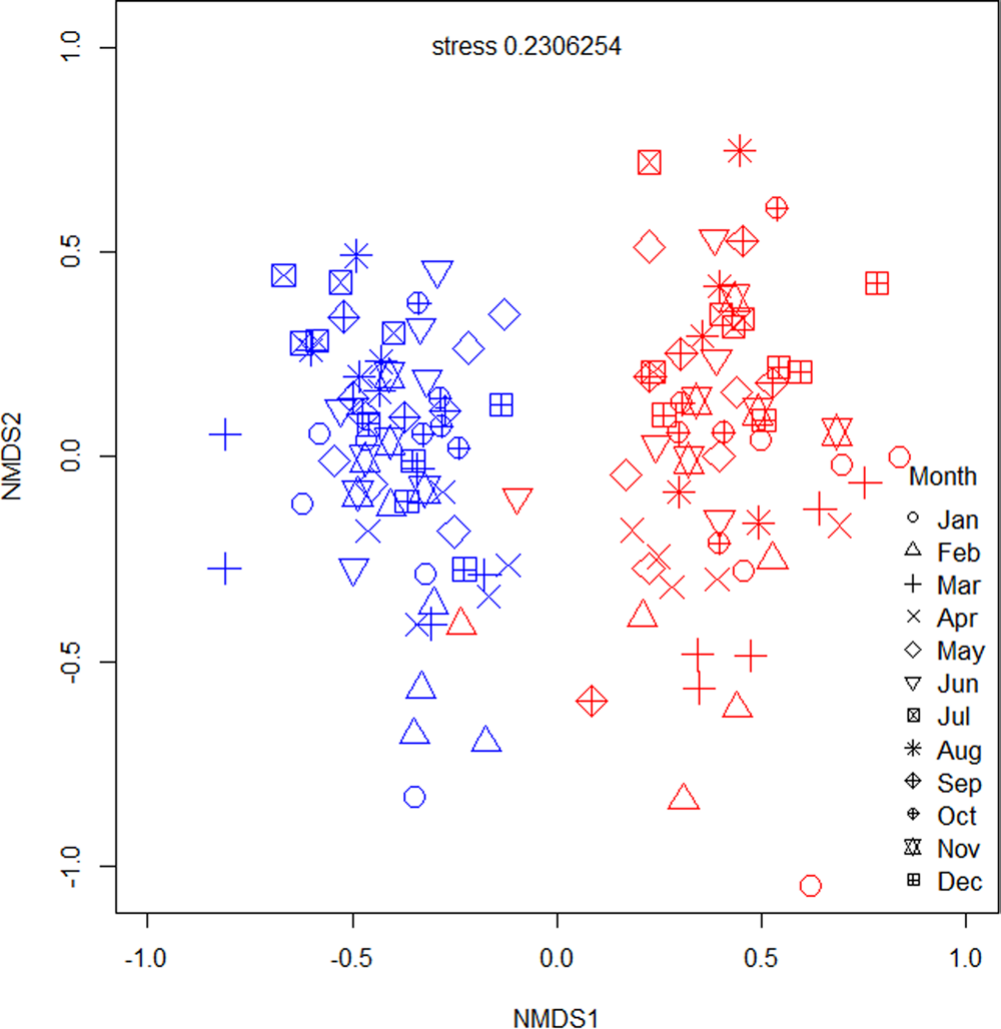
Ordination (NMDS) analysis of intra-annual differences in arthropod composition between forests and rubber for all species combined. Red symbols represent rubber plantation samples and blue symbols represent native forest samples. Each symbol represents the month in which the sample was collected.

**Figure 2:**
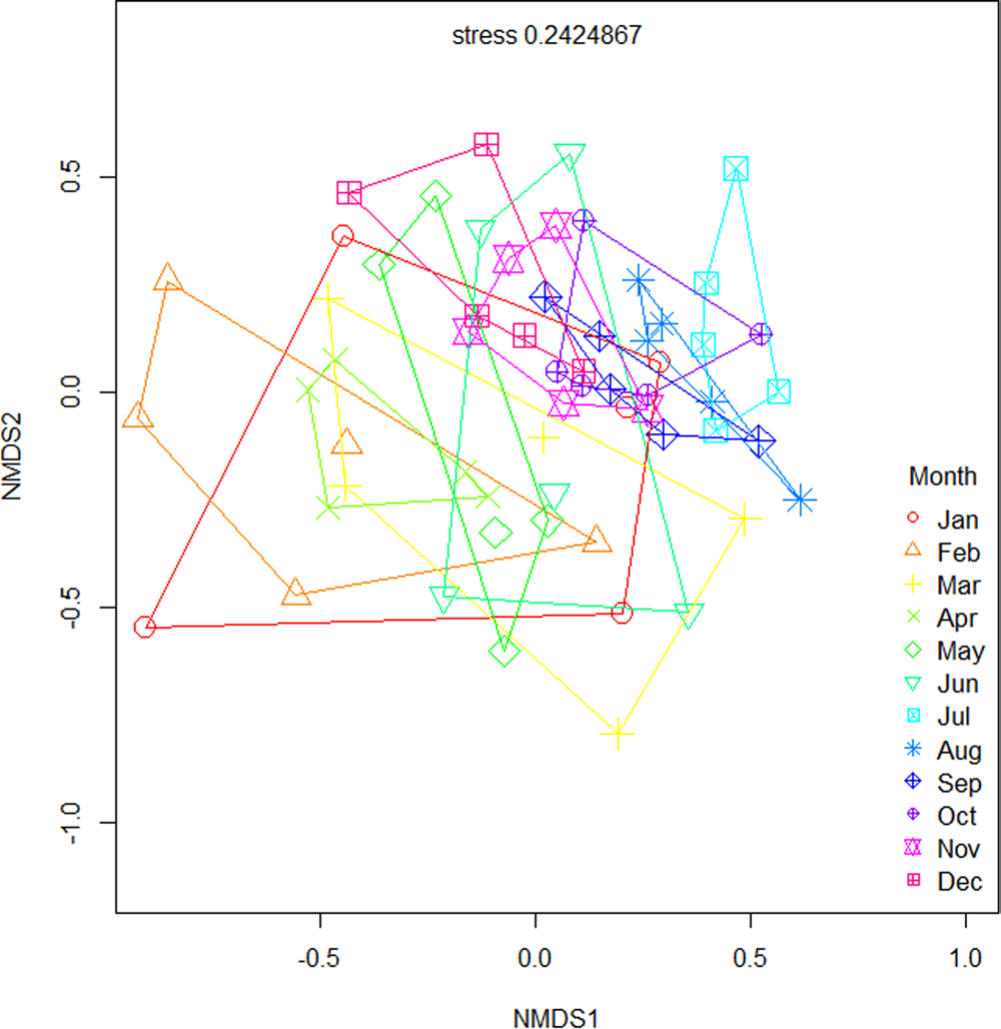
Non-metric multidimensional scaling (NMDS) analysis of intra-annual differences in arthropod species for native forests. Each symbol represents the month in which the sample was collected. Each ordihull represent the month in which the sample was collected and lines connect sites sampled during the same month.

**Figure 3:**
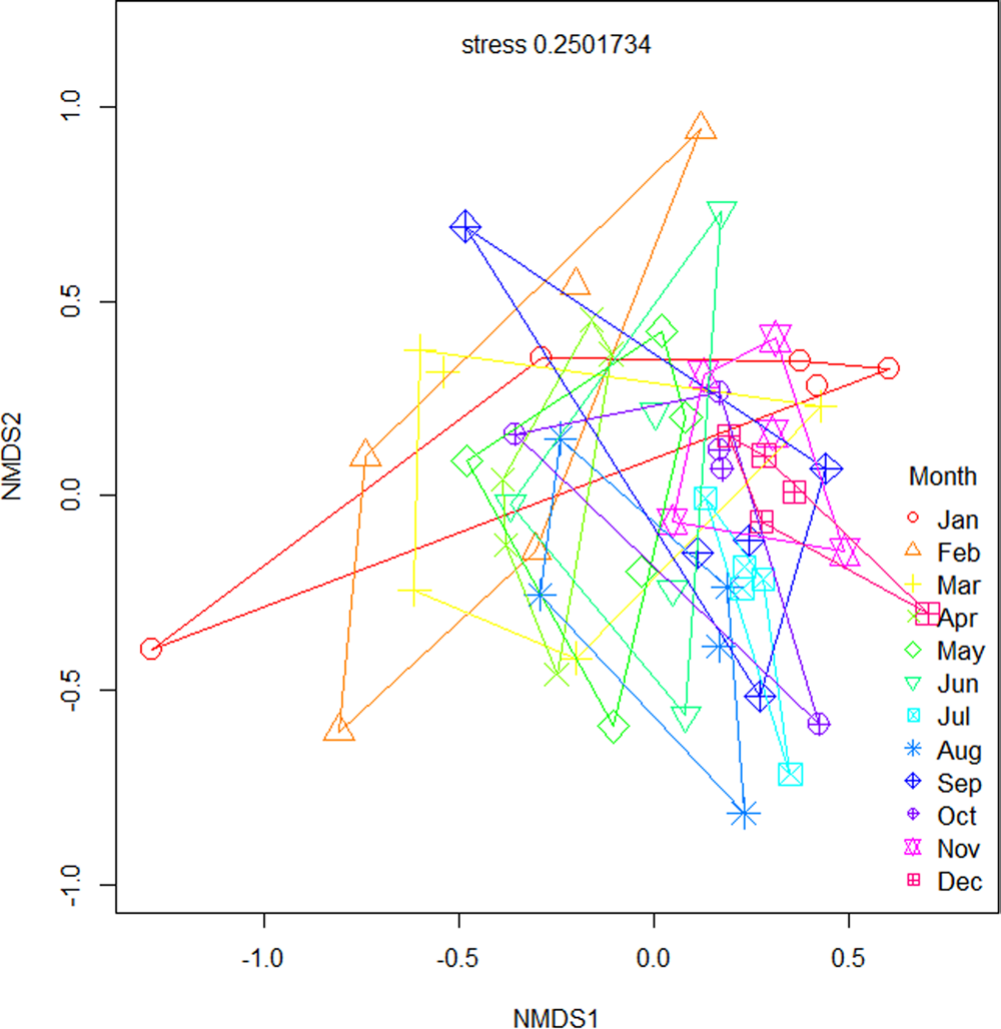
Non-metric multidimensional scaling (NMDS) analysis of intra-annual differences in arthropod species for rubber plantations. Each symbol represents the month in which the sample was collected. Each ordihull represent the month in which the sample was collected and lines connect sites sampled during the same month.

Ordination and repeated measures PERMANOVA revealed that native forests and rubber plantations have clearly distinct arthropod assemblages (F_1, 118_ = 16.0, p < 0.001), with communities from each habitat type forming distinct clusters throughout the year (Fig. 1). Each land-use type was therefore considered separately for intra-annual biodiversity assessments. We also detected significant intra-annual differences in species composition in both forests ((F_11, 48_ = 1.69, p < 0.001); Fig. 2, Table 1) and rubber ((F_11, 48_ = 1.66, p < 0.001; Fig. 3, Table 1). In general, species composition in the drier months from January to May was different from that in the wetter months from July to November, in both systems (Figs 2 and 3, Supplementary Information Table S1).

**Table 1:** Intra-annual patterns of species composition and richness in native forests and rubber plantations for all OTUs combined and for OTUs assigned to eight arthropod orders.

**Group**	**Land-use**	**Community differentiation**	**Species richness**
All OTUs	Forest	F_11, 48_ = 1.69, p < 0.001***	F_11, 48_ = 2.58, p < 0.011*
	Rubber	F_11, 48_ = 1.66, p < 0.001***	F_11, 48_ = 0.76, p = 0.676 ns
Araneae	Forest	F_11, 46_ = 0.75, p = 0.937 ns	F_11, 48_ = 3.83, p < 0.000***
	Rubber	F_11, 41_ = 2.30, p < 0.001***	F_11, 48_ = 1.57, p = 0.136 ns
Blattodea	Forest	F_11, 48_ = 1.33, p < 0.027*	F_11, 48_ = 5.48, p < 0.000***
	Rubber	F_11, 48_ = 1.64, p < 0.002**	F_11, 48_ = 2.80, p < 0.006**
Coleoptera	Forest	F_11, 48_ = 1.86, p < 0.001***	F_11, 48_ = 1.42, p = 0.192 ns
	Rubber	F_11, 48_ = 1.75, p < 0.001***	F_11, 48_ = 1.24, p = 0.287 ns
Diptera	Forest	F_11, 48_ = 1.82, p < 0.001***	F_11, 48_ = 3.40, p < 0.001**
	Rubber	F_11, 48_ = 1.48, p < 0.002**	F_11, 48_ = 0.41, p = 0.941 ns
Hemiptera	Forest	F_11, 48_ = 1.80, p < 0.001***	F_11, 48_ = 0.39, p = 0.951 ns
	Rubber	F_11, 48_ = 1.27, p < 0.033*	F_11, 48_ = 1.08, p = 0.390 ns
Hymenoptera	Forest	F_11, 48_ = 2.03, p < 0.001***	F_11, 48_ = 5.36, p < 0.000***
	Rubber	F_11, 48_ = 2.26, p < 0.001***	F_11, 48_ = 3.73, p < 0.000***
Isoptera	Forest	F_11, 48_ = 1.10, p = 0.234 ns	F_11, 48_ = 1.66, p = 0.111 ns
	Rubber	F_11, 45_ = 1.90, p < 0.001***	F_11, 48_ = 2.02, p < 0.046*
Orthoptera	Forest	F_11, 46_ = 1.18, p = 0.062 ns	F_11, 48_ = 1.10, p = 0.378 ns
	Rubber	F_11, 43_ = 1.10, p = 0.218 ns	F_11, 48_ = 0.68, p = 0.747 ns

Significance codes: 0 ‘***’ 0.001 ‘**’ 0.01 ‘*’ 0.05 ‘.’ 0.1 ‘’ 1. ns = not significant.

**Figure 4:**
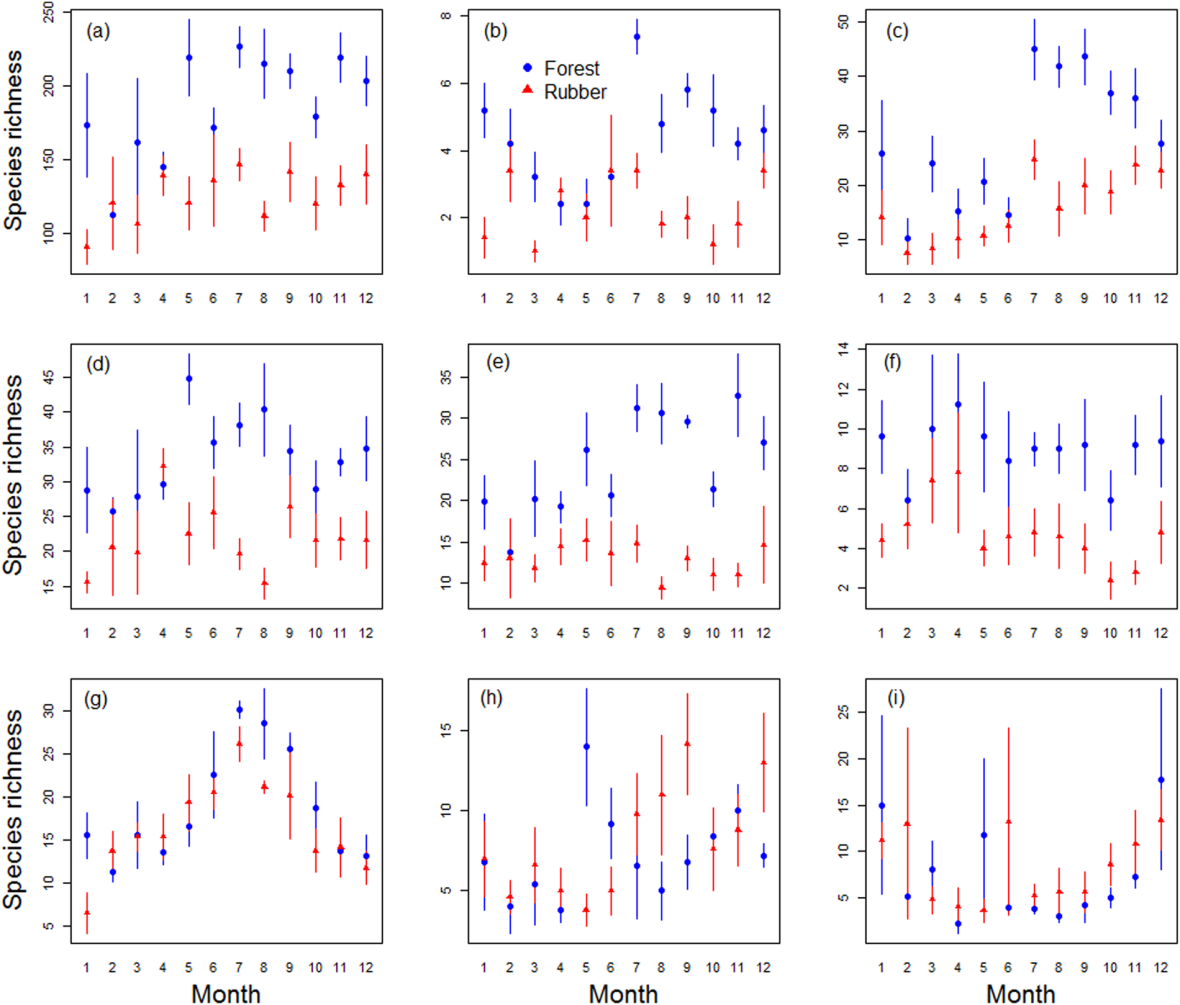
Mean arthropod species richness in forests (blue circles) and in rubber (red triangles) for; (**a**) all species combined, (**b**) Araneae, (**c**) Blattodea, (**d**) Coleoptera, (**e**) Diptera, (**f**) Hemiptera, (**g**) Hymenoptera, (**h**) Isoptera, and (i) Orthoptera. The lines represent standard errors of the mean. Richness was computed using 5 replicates per month. The numbers 1–12 on the x-axis represent January–December.

On average, forests were more species-rich than rubber plantations except in February (Fig. 4). Repeated measures ANOVA revealed significant intra-annual shifts in species richness in forests ((F_11, 48_ = 2.58, p < 0.01) but not in rubber (F_11, 48_ = 0.76, p = 0.676; Fig. 4, Table 1). Pairwise comparison using the Tukey’s Honest Significant Difference (TukeyHSD) test revealed that species richness in July, May and November were significantly higher than species richness in February for forests (Supplementary Information Table S2).

**Figure 5:**
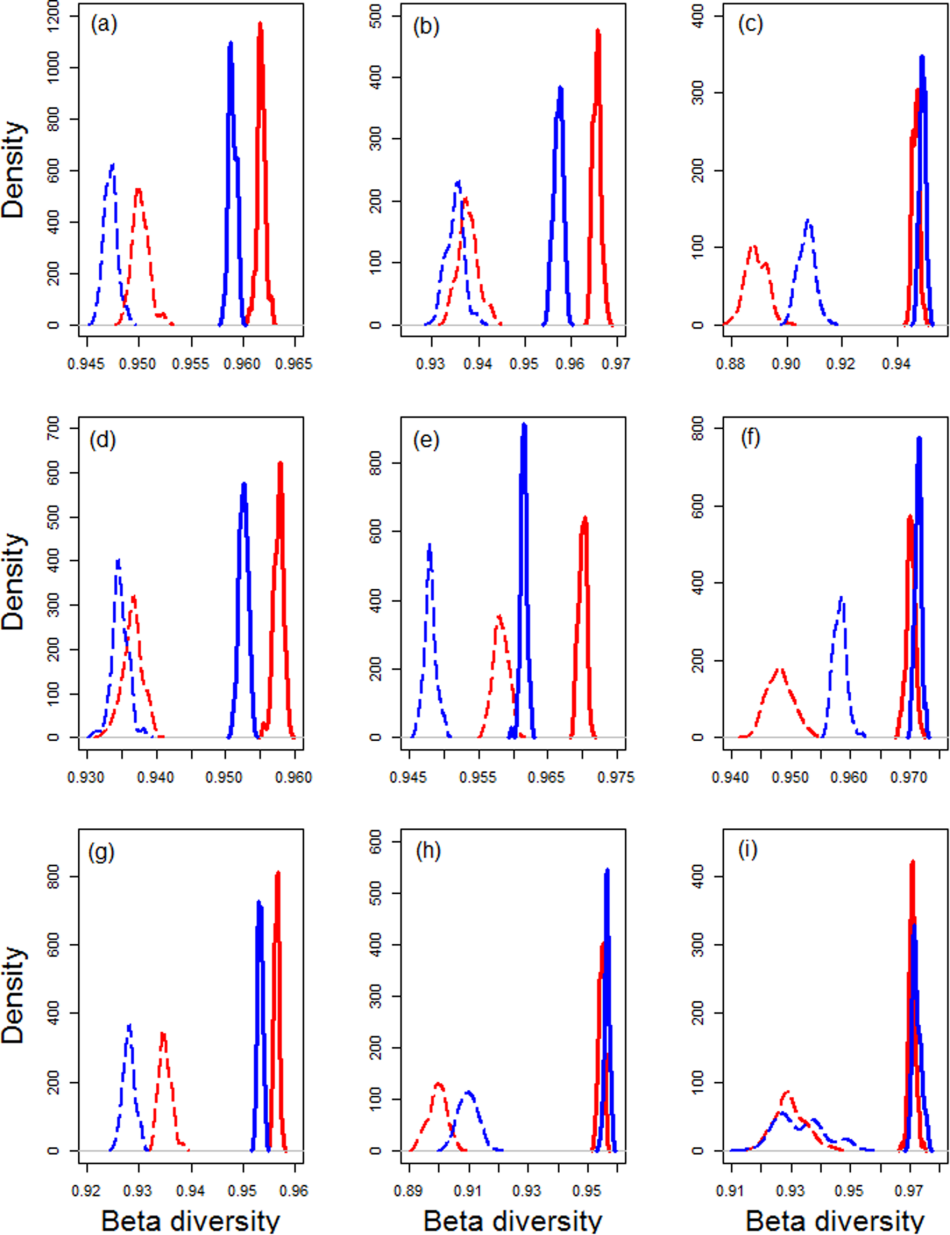
Total intra-annual β-diversity (smooth lines) and intra-annual species turnover (broken lines) for forests [blue color] and rubber [red color] sites. (**a**) All species combined, (**b**) Araneae, (**c**) Blattodea, (**d**) Coleoptera, (**e**) Diptera, (**f**) Hemiptera, (**g**) Hymenoptera, (**h**) Isoptera, and (**i**) Orthoptera. Values were computed using 100 bootstrap samples of 50 time pairs from each land-use type. Significant differences are detected when the peaks of the species turnover density plots do not overlap.

We detected higher levels of intra-annual species turnover (species replacement by new species not found in other months) in rubber than in forests (Fig. 5).

### Patterns of intra-annual variability across arthropod taxa

Orthoptera communities did not show any significant intra-annual differentiation in forests or rubber, but the patterns for all other taxa were more or less consistent with the general pattern for all species (Table 1). Seasonality affected species composition more strongly than species richness in Coleoptera, Diptera and Hemiptera in forest and rubber, and Araneae and Isoptera in rubber, implying that intra-annual variability resulted in species replacements rather than net losses and gains for these groups. For Blattodea and Hymenoptera, the effects of seasonality on both species composition and richness were strong, suggesting net losses and gains, as well as replacements.

Intra-annual patterns of species richness varied across taxonomic groups (Fig. 4, Table 1). Richness of Coleoptera, Hemiptera and Orthoptera did not show any significant intra-annual differences in either forests or rubber, while richness of Araneae and Diptera showed significant differences in forests but not in rubber (Table 1). Hymenoptera and Blattodea showed significant intra-annual differences in species richness in both land-use types, while Isoptera richness showed significant differences in rubber but not in forests (Table 1).

Intra-annual patterns of species turnover varied across taxonomic groups (Fig. 5). Species turnover was significantly higher in forests than in rubber for Blattodea, Hemiptera, and Isoptera, but significantly higher in rubber than in forests for Coleoptera, Diptera and Hymenoptera (Fig. 5). Araneae and Orthoptera did not show any obvious differences in turnover (Fig. 5).

### Correlates of arthropod community composition and richness

Intra-annual patterns of variation in environmental predictors (i.e. canopy openness, litter thickness, soil moisture, temperature and humidity) differed between forests and rubber (Table 2, Supplementary Information Fig. S1). The forest trees were almost all evergreen, but rubber trees in Xishuangbanna are briefly deciduous in January-February. Overall, rubber had a significantly higher canopy openness and temperature, and lower litter depth than forests, but soil moisture content (SMC) and humidity were similar (Table 2). Except for canopy openness in forests, all environmental variables showed significant intra-annual variation (Table 2), with temperature highest in June, soil moisture in August, and humidity in November-December, in both land-use types (Supplementary Information Fig. S1). Litter depth was highest in January-February in rubber and March-May in forest. Canopy openness in rubber was also highest in January-February (Supplementary Information Fig. S1).

**Table 2:** Patterns of intra-annual environmental variability between forests and rubber computed using repeated measures Analysis of Variance (ANOVA).

Variable	Land-use variability	Intra-annual variability	
	Forest vs Rubber	Forest	Rubber
Canopy openness (%)	F_1, 8_ = 21.25, p < 0.001**	F_11, 44_ = 0.86, p = 0.579 ns	F_11, 44_ = 63.76, p < 0.000***
Litter depth (cm)	F_1, 8_ = 7.32, p < 0.026*	F_11, 44_ = 13.44, p < 0.000***	F_11, 44_ = 53.21, p < 0.000***
Soil moisture content (%)	F_1, 8_ = 0.18, p = 0.680 ns	F_11, 44_ = 36.48, p < 0.000***	F_11, 44_ = 19.59, p < 0.000***
Temperature (°C)	F_1, 8_ = 25.00, p < 0.001**	F_11, 44_ = 186.70, p < 0.000***	F_11, 44_ = 176.70, p < 0.000***
Humidity (%)	F_1, 8_ = 0.01, p = 0.937 ns	F_11, 44_ = 57.39, p < 0.000***	F_11, 44_ = 47.41, p < 0.000***

Significance codes: 0 ‘***’ 0.001 ‘**’ 0.01 ‘*’ 0.05 ‘.’ 0.1 ‘’ 1. ns = not significant.

All five environmental predictors (i.e. canopy openness, litter thickness, soil moisture, temperature and humidity) were strongly associated with intra-annual patterns of arthropod composition in both forests and rubber (Table 3). The influence of environmental variables on intra-annual compositional shifts varied across arthropod taxa (Supplementary Information Table S3).

**Table 3:** Model fit parameters from Canonical Correspondence Analysis (CCA) gradient analysis of arthropod community composition in forests and in rubber.

**Native forests**					**Rubber plantations**			
**CCA model**	**Df**	***χ*** ^**2**^	**F**	**Pr(>F)**	**Df**	***χ*** ^**2**^	**F**	**Pr(>F)**
Model	5	1.18	1.29	0.001***	5	1.42	1.39	0.001***
Residual	54	9.90			54	11.02		
**Axis**								
CCA1	1	0.31	1.70	0.001***	1	0.35	1.69	0.003**
CCA2	1	0.25	1.36	0.002**	1	0.34	1.68	0.001***
CCA3	1	0.22	1.21	0.041*	1	0.27	1.33	0.021*
CCA4	1	0.21	1.18	0.032*	1	0.25	1.23	0.046*
CCA5	1	0.18	1.01	0.397	1	0.21	1.03	0.355
Residual	54	9.90			54	11.02		
**Variables**								
Canopy openness	1	0.24	1.31	0.001***	1	0.31	1.51	0.001***
Litter thickness	1	0.25	1.34	0.001***	1	0.24	1.17	0.043*
Soil moisture	1	0.25	1.36	0.001***	1	0.33	1.60	0.001***
Temperature	1	0.23	1.25	0.001***	1	0.26	1.28	0.003**
Humidity	1	0.23	1.23	0.002**	1	0.28	1.39	0.001***
Residual	54	9.90			54	11.02		

The significance of the CCA model, CCA axes and environmental variables was tested using 999 permutations and only variables with p < 0.05 were considered to have significant effects. Significance codes: 0 ‘***’ 0.001 ‘**’ 0.01 ‘*’ 0.05 ‘.’ 0.1 ‘’ 1.

Generalized linear mixed models revealed that temperature and humidity were most strongly positively associated with species richness in forests (Table 4). None of the tested environmental variables significantly influenced species richness in rubber (Table 4). The influence of environmental variables on intra-annual patterns of species richness varied across arthropod taxa (Supplementary Information Table S4).

**Table 4:** Factors that influence arthropod species richness in forests and in rubber computed using generalized linear mixed-effects regression with random effects for site.

**Native forests**					**Rubber plantations**			
**Random effects:**								
Groups	Variance	Std.Dev.			Variance	Std.Dev.		
Site (n = 5)	0.16	0.39			0.16	0.40		
Residuals	3.17	1.78			3.19	1.78		
**Fixed effects:**								
	**Estimate**	**Std.Error**	**t value**	**p-value**	**Estimate**	**Std.Error**	**z value**	**p-value**
(Intercept)	13.49	0.29	46.5		11.06	0.29	38.01	
Canopy openness	0.50	0.27	1.86	0.062 ·	0.26	0.42	0.61	0.542
Litter thickness	−0.19	0.36	−0.52	0.604	−0.57	0.38	−1.48	0.138
Soil moisture	−0.26	0.32	−0.81	0.416	−0.06	0.35	−0.18	0.855
Temperature	0.59	0.28	2.14	0.033*	0.35	0.41	0.85	0.396
Humidity	1.04	0.40	2.60	0.009**	0.07	0.43	0.16	0.873

Model <− glmer (Richness ~ Canopy openness + Litter thickness + Soil moisture content + Temperature + Humidity + (1|Plot), method = “REML”). Significance codes: 0 ‘***’ 0.001 ‘**’ 0.01 ‘*’ 0.05 ‘.’ 0.1 ‘’ 1.

### Number of times to sample per year

**Figure 6:**
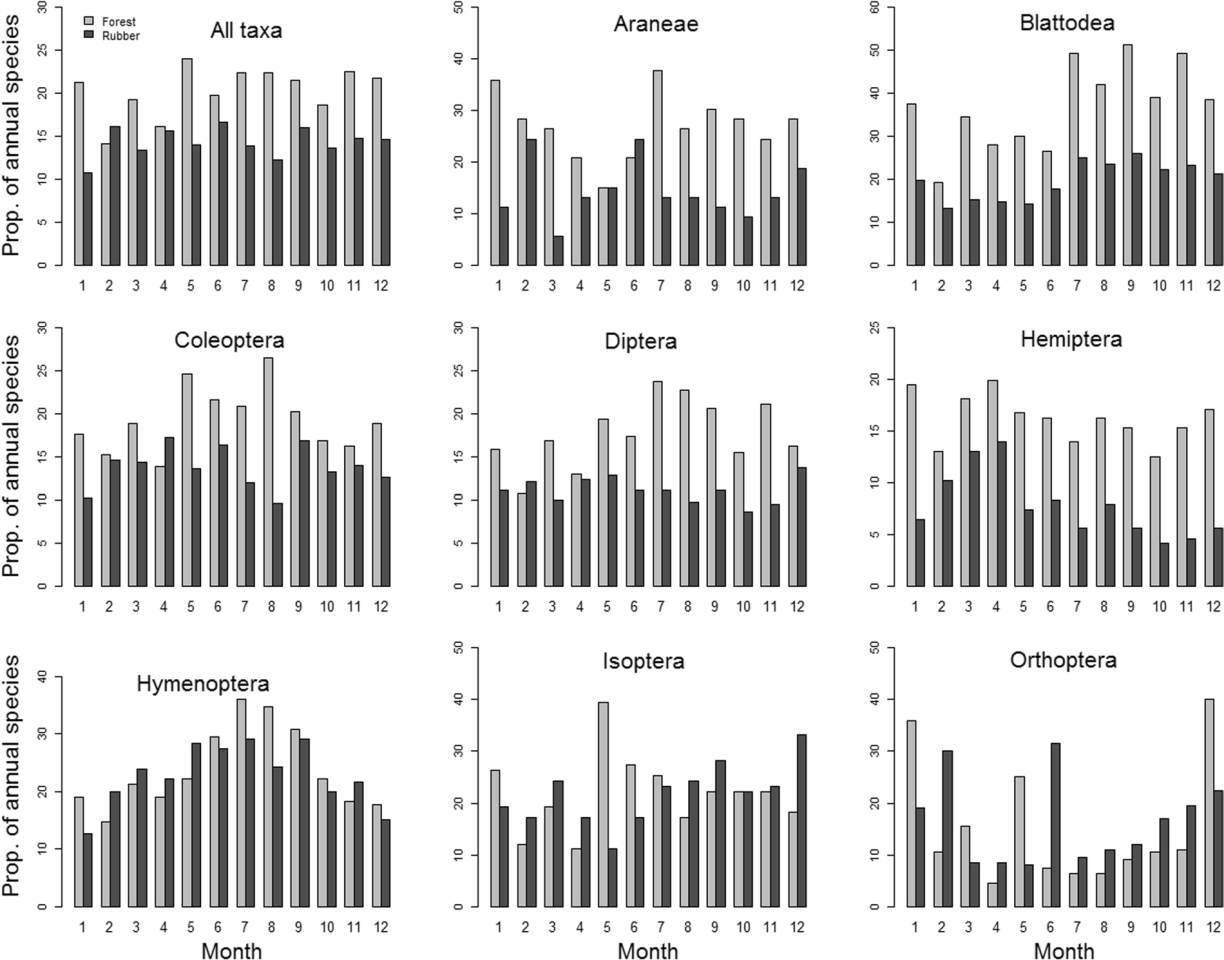
The proportion (%) of annual arthropods detected in each month in forests and rubber. Data is presented for all taxa combined and for individual taxonomic groups.

We found that sampling once a year only captured 14–24% and 11–17% of the total number of arthropods found in the 12 monthly samples in forests and rubber, respectively (Fig. 6). However, single-sample efficiency varied considerably across arthropod taxa, from 5% for Orthoptera to 51% for Blattodea in forests, and from 4% for Hemiptera to 32% for Orthoptera in rubber (Fig. 6).

**Figure 7:**
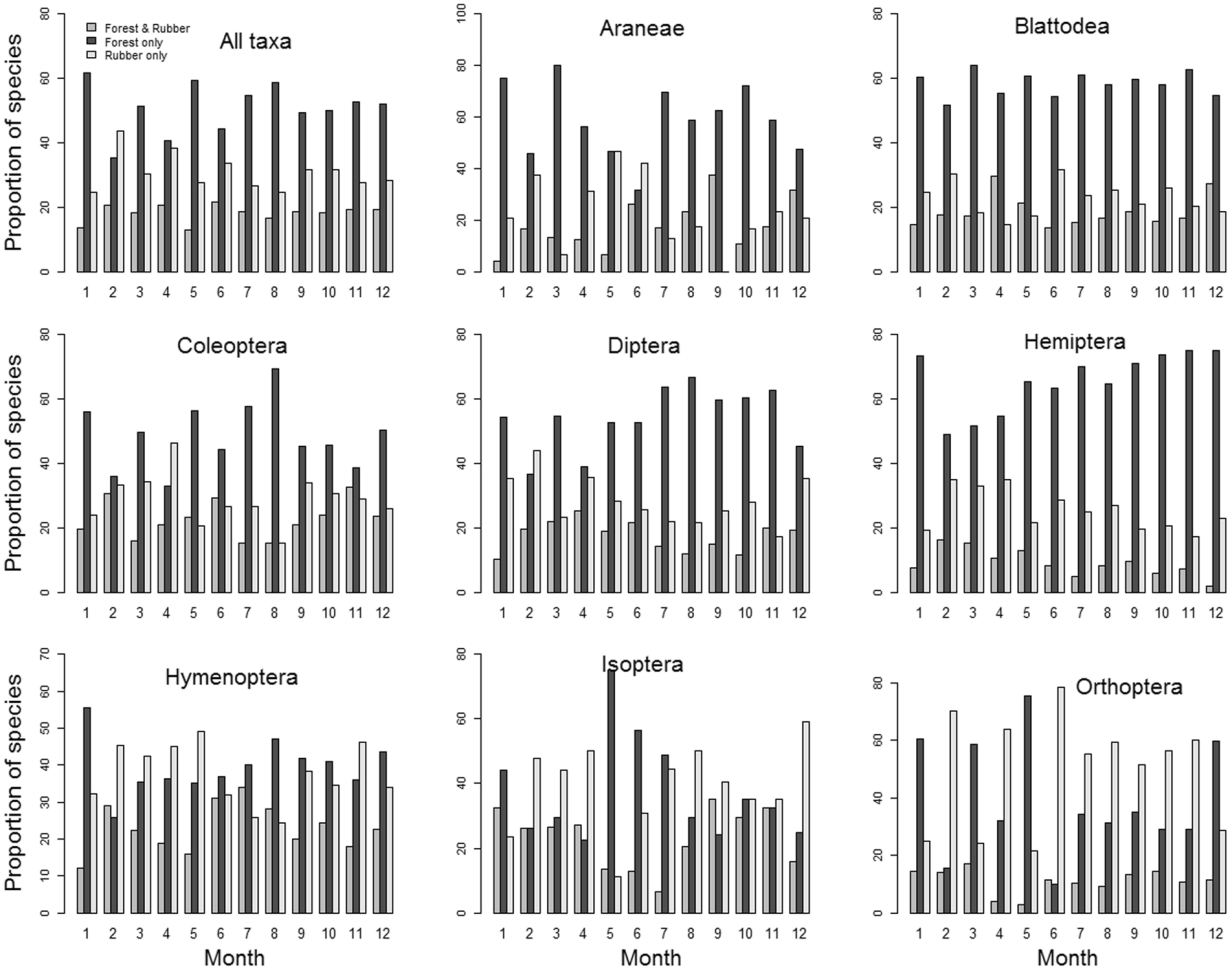
The proportion (%) of monthly arthropod species that were exclusively detected in forests (Forest only), exclusively detected in rubber (Rubber only) and detected in both forests and rubber (Forest & Rubber). Data is presented for all taxa combined and for individual taxonomic groups.

We found that forests harbor a relatively higher percentage of unique arthropod species (36–62% of species not found in rubber) than rubber (25–44% of species not found in forests), except in February, but this pattern was not consistent across arthropod taxa (Fig. 7). Forests and rubber shared 13–22% of all arthropod species captured each month and always <40% for any arthropod taxon (Fig. 7).

Based on analysis of species accumulation curves using custom R scripts, sampling twice a year (May and August) captured 38%, sampling thrice a year (May, August and November) captured 46%, while sampling four times a year (January, May, August and November) captured 52% of the total diversity in native forests (Supplementary Information Fig. S2). In rubber plantations, sampling twice a year (February and September) captured 27%, sampling thrice a year (February, April and September) captured 34%, while sampling four times a year (February, April, July and September) captured 40% of the total diversity (Supplementary Information Fig. S3).

## Discussion

Native forests and rubber plantations had very different arthropod assemblages throughout the year (<25% of species shared in any month), with forests more species-rich than rubber in all months except February. This pattern might be related to February being in the middle of the cold dry season, when arthropod species are least active. Species composition in the drier months (from January to May) was different from that in the wetter months (from July to November) in both forests and rubber, in agreement with studies in other parts of the seasonal tropics^14–17^. Conversion of native forests to rubber plantations also reduced macroinvertebrate diversity in lowland Sumatra, although the impact was less than with conversion to oil palm^18^. Our forests were more species-rich in every taxonomic group except Isoptera and Orthoptera, where rubber was richer in more than half the months. Most orthopterans are open-habitat species and constitute important herbivores in many terrestrial ecosystems. Orthoptera species richness in a given habitat is mediated by the quality and quantity of food resources (e.g. species richness and biomass of grasses) available^19^. Rubber plantations have relatively higher canopy openness and this promotes understorey vegetation emergence. Furthermore, management regimes (e.g. manual cutting and fertilizer application) also help maintain high grass and forb diversity by limiting single species dominance. Though there is probably more dead plant matter in rubber plantations than forests, that would not lead to higher diversity, which is more likely to result from more diverse dead plant matter. The high species richness of Isoptera in rubber plantations was unexpected, given the lower diversity of food resources for detritivores, and requires further study.

We detected higher levels of intra-annual species turnover (species replacement by new species not found in other months) in rubber than in forests, which may reflect the greater seasonality in rubber of potential environmental drivers. In particular, canopy openness and litter depth both increased sharply during the brief deciduous period in February. Rubber plantations also experience periodic disturbances throughout the year from rubber tappers, understorey clearance with herbicides or manual cutting, and fertilizer and pesticide applications. However, there was little seasonality in species richness, showing that the high species turnover in rubber reflects species replacements without overall losses or gains throughout the year.

The most novel and striking finding of this study is the high rate of seasonal turnover in species composition, which has implications for both sampling and ecosystem functions. The Winkler method of extracting arthropods from litter used in this study depends on their active movement, so inactive stages (eggs, pupae) would not have been sampled. Alternatively, active individuals could have moved from the sampled volume. It is unlikely that any species migrates seasonally more than a few meters, but they may move into the soil below the litter layer, or retreat to favorable microsites, or simply be too rare to detect. Whatever the cause of species turnover, sampling only once underestimates the total diversity of the fauna by a factor of four to nine. Sampling at least four times over the year was necessary to capture half the total fauna. Given that species composition was different between land-use types for all months except February, it is worth mentioning that capturing the total diversity and turn over, over 4+ months, although highly relevant and important for understanding functional differences, is not always necessary if the goal is to simply demonstrate that communities differ between the two land-use types (i.e. biomonitoring).

The limitations of the reference barcode database preclude a detailed analysis of the functional implications of high species turnover, but it is possible that this could contribute to the large seasonal variation in litter decomposition recorded in our study area^3^. Alternatively, arthropod species replacements could buffer leaf litter decomposition rate against environmental seasonality. For instance, in assemblages with a certain combination of species, litter decomposition might not be affected by temperature seasonality when compared with constant temperature conditions. This would be a potentially fertile area for future research.

Finally, this study demonstrates once again the utility of DNA barcodes for working with phylogenetically diverse arthropod communities^10^. It would not have been possible to sort and identify the thousands of specimens spread across multiple orders by traditional means. This study also demonstrates the limitations of working with molecular OTUs without being able to assign genus- or species-level taxonomy and thus make use of the growing literature on arthropod ecologies. Improving the barcode database needs to be a higher priority in tropical areas.

## Conclusions

The litter layer of tropical forests supports a significant fraction of total arthropod diversity and on-going conversion of tropical forests to agricultural monocultures constitutes the most important threat to biodiversity and associated ecosystem services. We found large intra-annual shifts in community composition in both forests and rubber, with the patterns varying across taxa. Our results confirm existing evidence that tropical litter arthropod communities are very sensitive to seasonality and demonstrate that sampling at only one time of the year captures only a subset of the total community. The study also demonstrates the utility of DNA barcodes for investigating phylogenetically diverse arthropod communities and the urgent need for improvements in the barcode database.

## Materials and Methods

### Study area and site selection

Xishuangbanna (22°00′N 100°48′E; henceforth XSBN) is a prefecture in Yunnan province, in southwest China, on the northern margin of tropical Southeast Asia within the Indo-Burma biodiversity hotspot^20,21^. It covers an area of 19,690 km^2^ and borders on Myanmar to the southwest and on Laos to the south and southeast. The topography is mountainous, with altitudes ranging from 480 to 2,429 m above sea level. XSBN experiences a tropical monsoon climate with a hot, wet season (May to October) with 80% of the annual rainfall, a foggy cool season (November to February), when morning fog reduces evapotranspiration, ‘fog drip’ moistens the litter layer, and leaves wetted by fog can take up water directly^22^, and a dry hot season (March to April), when water stress reaches a maximum. Menglun (101.15°E–101.43°E, 21.81°N–22.00°N) is a typical township representative of the environmental and socio-economic conditions of XSBN and covers an area of 335 km^2^ with altitudes ranging from 540 to 1,400 m^23^. Mean annual temperature in Menglun town is 21.5 °C and mean annual rainfall is 1563 mm. The last three decades have witnessed a massive expansion of rubber plantations at the expense of tropical forests^24,25^, leaving the remaining forests as fragments of varying sizes in the landscape^26^. The rubber plantations in our study area are monocultures of the Neotropical tree, *Hevea brasiliensis*, which are cleared of understorey vegetation, tapped for latex every other day from March to November, and treated with pesticides, fungicides, and herbicides.

Five matched pairs of sites within native forests and adjacent rubber plantations were selected from an existing fragmentation project^26^ within a 20 km-diameter circle around the Xishuangbanna Tropical Botanical Garden (21°55′N 101°15′E) in Menglun (Supplementary Information Fig. S4). Matched pairs were selected to be close to each other to minimize differences in elevation and soil properties (Supplementary Information Table S5).

Permission to collect samples from the native forest sites was obtained from the Yunnan Provincial Bureau of Forestry (Research permit: [2014] No. 13) while permission to collect samples from the rubber plantation sites was obtained from the plantation owners (verbal consent). All procedures and methods were performed in accordance with the approved guidelines.

### Sample collection and preparation

In each site, nine 1 × 1 m^2^ quadrats (placed 10 m apart; one in the middle and two each in north, east, west and south directions) were established in each land-use type (Supplementary Information Fig. S5). Within each quadrat, all leaf litter and loose humus were collected into a large polythene bag and sieved through a wire mesh (0.8 cm × 0.8 cm) to remove larger litter materials. The sifted litter of nine quadrats of each site was combined, placed in a polythene bag, taken to the laboratory and subjected to 72 hours Winkler incubation^27^. Sampling was done monthly from January-December 2014, and quadrats of the preceding month’s sampling were placed within 1–2 m of the current month’s quadrats (Supplementary Information Fig. S5). The following environmental variables were also recorded monthly in each land-use type at each site from January-December 2014;

1. *Canopy openness*; from nine hemispherical (true-color fisheye) photographs using the Gap Light Analyzer (GLA) version 2.0 software.
2. *Litter thickness*; at three points within each of the nine 1 × 1 m^2^ quadrats (27 measurements per site) with a ruler.
3. *Soil moisture content (SMC)*; from 200 g of soil collected from the nine 1 × 1 m^2^ quadrats using a corer (10 cm depth), homogenized, oven-dried to constant weight and reweighed.
4. *Temperature*; at 30-minutes intervals over the whole year using one iButton Hygrochron data logger (https://www.maximintegrated.com) installed at 0.5 m above the ground in the middle of each site.
5. *Humidity*; at 30-minutes intervals over the whole year using the same iButton Hygrochron data loggers as above.

### DNA extraction, amplification and sequencing

In order to keep the final DNA quantity similar across individual arthropods, we used two legs from all individuals with body length ≥5 mm and the whole bodies of everything smaller. Bulk samples were subsequently freeze-dried using liquid nitrogen, ground, and homogenized using a mortar and pestle. Genomic DNA was extracted using the DNeasy Tissue Kit (QIAGEN; Hilden, Germany; protocol for animal tissues) according to the manufacturer’s instructions.

We amplified a 400 bp fragment of the mitochondrial cytochrome c oxidase subunit I gene (COI) corresponding to the standard DNA barcoding region of most arthropods^10^ using the universal primer pair MhemF^28^ and dgHCO2198^29^. PCR was carried out in a total volume of 50 µL using 10 ng DNA, 5.0 µL 10× PCR buffer, 0.5 mM dNTPs, 2.5U Platinum Taq (TaKaRa Biosystems, Ohtsu, Shiga, Japan) 0.5 µL of each of forward and reverse primers. PCR cycling conditions were 94 °C for 3 min, 5 cycles of 94 °C for 30 s; 45 °C for 20 s; 72 °C for 30 s; then 20 cycles of 94 °C for 20 s; 55 °C for 20 s; 72 °C for 30 s and finally 72 °C for 5 min. PCR products were size-verified by gel electrophoresis. Purified PCR products were sequenced in both directions with 2 × 300 cycles using the MiSeq Reagent Kit v3 (Illumina, Inc., 2015) as per manufacturer’s instructions and the same PCR primers.

### Read preparation and OTU clustering

Pair-end reads were merged using the Fast Length Adjustment of SHort reads (FLASH v1.2.7) software^30^. Merged reads were quality filtered by applying the expected error (predicted by Phred (Q) scores) filtering technique and a maximum expected error threshold of 0.50^31^. Reads that did not meet this quality criteria (expected errors >0.5) were discarded. OTU recovery was performed using the UPARSE algorithm^32^. Quality filtered reads were dereplicated, sorted by abundance and all singleton clusters were discarded. This process does not affect the final results because successfully amplified DNA should be found in at least two copies^33^. Unique reads were clustered into OTUs with a minimum similarity of 97%. This step also discards reads that have chimeric models built from more abundant sequences^32^. An OTU table (OTU x site matrix) was constructed (Supplemental Dataset 1). The taxonomy of each OTU was predicted using the Ultra-fast global alignment search for high-identity top hits (usearch_global) tool^34^. This alignment search tool checks a reference database for high-identity hits to one or more reference sequences (“targets”) using word counts to prioritize the database search. Target sequences are compared to the query in order of decreasing unique word count. For taxonomy assignment, we downloaded and used a database of 3,573,622 Arthropoda COI sequences from the Barcode of Life Database (BOLD^35^; date assessed 13/06/2017). We used a recommended nucleotide top hit identity cutoff of 75% for which USEARCH is effective for nucleotides. All analyses were performed using freely available 32-bit USEARCH v10.0.240 for Linux (https://www.drive5.com/usearch/download.html, date assessed 14/06/2017).

### Quality filtering and UPARSE OTU recovery

We obtained a total of 2,551,110 quality filtered reads of which 1,089,076 (42.7%) were unique reads and 950,836 (37.3%) were singletons. The unique reads were clustered into 3,099 OTUs and 884 chimeras were detected during the OTU recovery step. Of the 3,099 OTUs recovered, 213 could not be successfully assigned to any arthropod order (Supplementary Dataset 2). We therefore removed these unassigned OTUs from downstream analyses. The OTU sequences were deposited in the NCBI database under GenBank nucleotide sequence accession numbers MF737646 - MF740744. Recent studies in a range of arthropod families suggest that OTUs delimited by COI barcodes with a minimum similarity of 97% will mostly be equivalent to morphological species, although the degree of congruence varies between orders, spatial scales, and methods^36–38^. The limitations of the reference barcode database make it impossible to check this assumption directly with our own data, but OTUs will hereafter be referred to as species for simplicity.

### Statistical analysis

We examined whether differences in total number of reads per sample affected OTU richness estimates by comparing conventional diversity and evenness metrics (number of OTUs, Chao1, Shannon and Simpson) with rarefied OTU richness. Rarefied OTU richness was calculated by subsampling the OTU table for the same number of reads (13,665) across all samples^39^.

We found significant positive relationships between Chao1 and rarefied species richness [(t value = 45.43), p < 0.001, adj r^2^ = 0.95] and between observed number of species and rarefied species richness [(t value = 124.60), p < 0.001, adj r^2^ = 0.99] (Supplementary Information Fig. S6). The relationships between Simpson and rarefied species richness [(t value = −2.79), p < 0.01, adj r^2^ = 0.05] and between Shannon and rarefied species richness [(t value = 6.70), p < 0.001, adj r^2^ = 0.27] were significant but weak (Supplementary Information Fig. S6). We therefore used observed number of species/OTUs (richness) for all analyses.

Nonmetric multidimensional scaling (NMDS) ordinations were conducted to examine whether the composition of arthropod communities in forests and in rubber changed over the course of the year^39^. We used Constrained Correspondence Analysis (a.k.a. canonical correspondence analysis, CCA) to determine which environmental factors were most strongly associated with these changes^39^. The significance of the CCA model, CCA axes and environmental variables was tested using 999 permutations and only models, axes and variables with p < 0.05 were considered to be statistically significant^39^. The OTU table was transformed into a presence-absence matrix prior to NMDS and CCA analyses.

As multiple measurements were made on the same sites over a year, we used repeated measures permutational multivariate analysis of variance (PerMANOVA) using a distance matrix and sites as strata to test for intra-annual variation in species composition^39^. When a significant effect was found, pairwise contrasts were computed to determine which of the month-to-month pairs were significantly different in assemblage composition. P values were adjusted for multiple comparisons using the “fdr” method^40^.

We analyzed intra-annual differences in mean species richness using repeated measures Analysis of variance (ANOVA). This method accounts for replicated sampling in the same site. When a significant effect was found, multiple comparison tests were conducted to determine which of the month-to-month pairs were significantly different.

As multiple measurements were made on the same sites over a year, generalized linear mixed models with random effects for site were used to determine which explanatory variables were strongly associated with observed patterns of species richness^41^. Prior to regression analysis, all predictors were standardized to zero mean and unit variance to satisfy the assumptions of the residuals conforming to a normal distribution. Predictors that did not meet the assumption of normality were square root transformed. Environmental predictors were also tested for multicollinearity before using them in constrained ordination and general linear mixed models. All pairwise correlation coefficients were <0.65 (Supplementary Information Fig. S7).

We computed beta (β)-diversity in forest and in rubber as pairwise Sørensen and Simpson indices using the betapart 1.3.package^42^. These analyses were done using 50 random month-to-month pairs from the 66 possible pairs for each land-use type (i.e. Jan-Feb, Jan-Mar, Jan-Apr … etc), then resampling 100 times. The between-months β-diversity was decomposed into its turnover (species replacement from month to month) and nestedness (species gain/loss between months) components^42^. Significant differences are detected when the peaks of the species turnover density plots do not overlap^42^.

To determine how many times a year it is necessary to sample in order to assess arthropod biodiversity in a seasonal tropical climate, we used species accumulation curves to extract richness (number of OTUs) for all combinations of two, three and four months, and recorded the combination with the highest richness^39^. All analyses were conducted on all OTUs combined and on subsets of OTUs assigned to eight arthropod orders (Araneae, Blattodea, Coleoptera, Diptera, Hemiptera, Hymenoptera, Isoptera and Orthoptera). All analyses were performed using the R statistical software^43^.

## Electronic supplementary material

Dataset 2

Dataset 1

Supplementary Information
