# Supplementary material for: Seasonal changes in the diversity and composition of the litter fauna in native forests and rubber plantations
Source: Sci Rep. 2018 Jul 6;8:10232. doi: 10.1038/s41598-018-28603-7 (PMC6035245; doi:10.1038/s41598-018-28603-7)
Supplement: Supplementary file 3 — Supplementary Information [file 41598_2018_28603_MOESM3_ESM.docx]

**Seasonal changes in the diversity and composition of the litter fauna in native forests and rubber plantations**

Kingsly C. Beng^1*^; Richard T. Corlett^1^; Kyle W. Tomlinson^1^

^1^Center for Integrative Conservation, Xishuangbanna Tropical Botanical Garden, Chinese Academy of Sciences, Menglun, Mengla, Yunnan, 666303, China.

*****Corresponding author


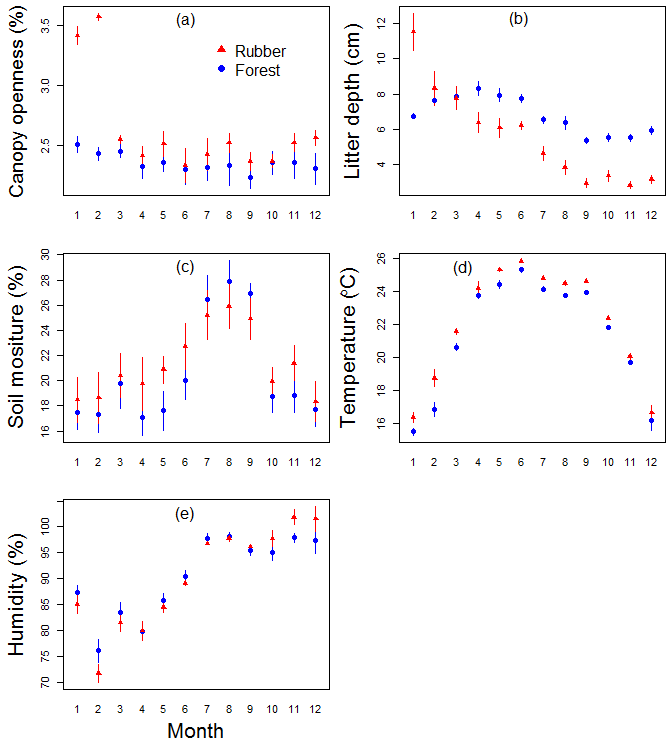


**Figure S1**. Intra-annual patterns of environmental variability in forests and in rubber showing means ± standard error. *Canopy openness* (log transformed) was measured from nine hemispherical (true-color fisheye) photographs using the Gap Light Analyzer (GLA) version 2.0 software. *Litter depth* was measured at three points within each of the nine 1 x 1 m^2^ quadrats (27 measurements per site) with a ruler. *Soil moisture content (SMC)* was measured from 200 g of soil collected from the nine 1 x 1 m^2^ quadrats using a corer (10 cm depth), homogenized, oven-dried to constant weight and reweighed. *Temperature* and *Humidity* were measured at 30-minutes intervals over the whole year using iButton Hydrochron data loggers installed at 0.5 m above the ground. The numbers 1 - 12 on the x-axis represent January – December.

**Figure S2**. Species accumulation curve for native forests

Best two combinations:

May-July: 1085 of 2885 species (37.6%)

May-August: 1086 of 2885 species (37.6%)

Best three combinations:

May-August-November: 1327 of 2885 species (46.0%)

Best four combinations:

March-May-August-November: 1508 of 2885 species (52.3%)

May-July-August-November: 1509 of 2885 species (52.3%)

January-May-August-November: 1510 of 2885 species (52.3%)

**Figure S3**. Species accumulation curve for rubber plantations.

Best two combinations:

February-September: 779 of 2885 species (27.0%)

Best three combinations:

February-April-September: 987 of 2885 species (34.2%)

Best four combinations:

February-April-July-November: 1142 of 2885 species (39.6%)


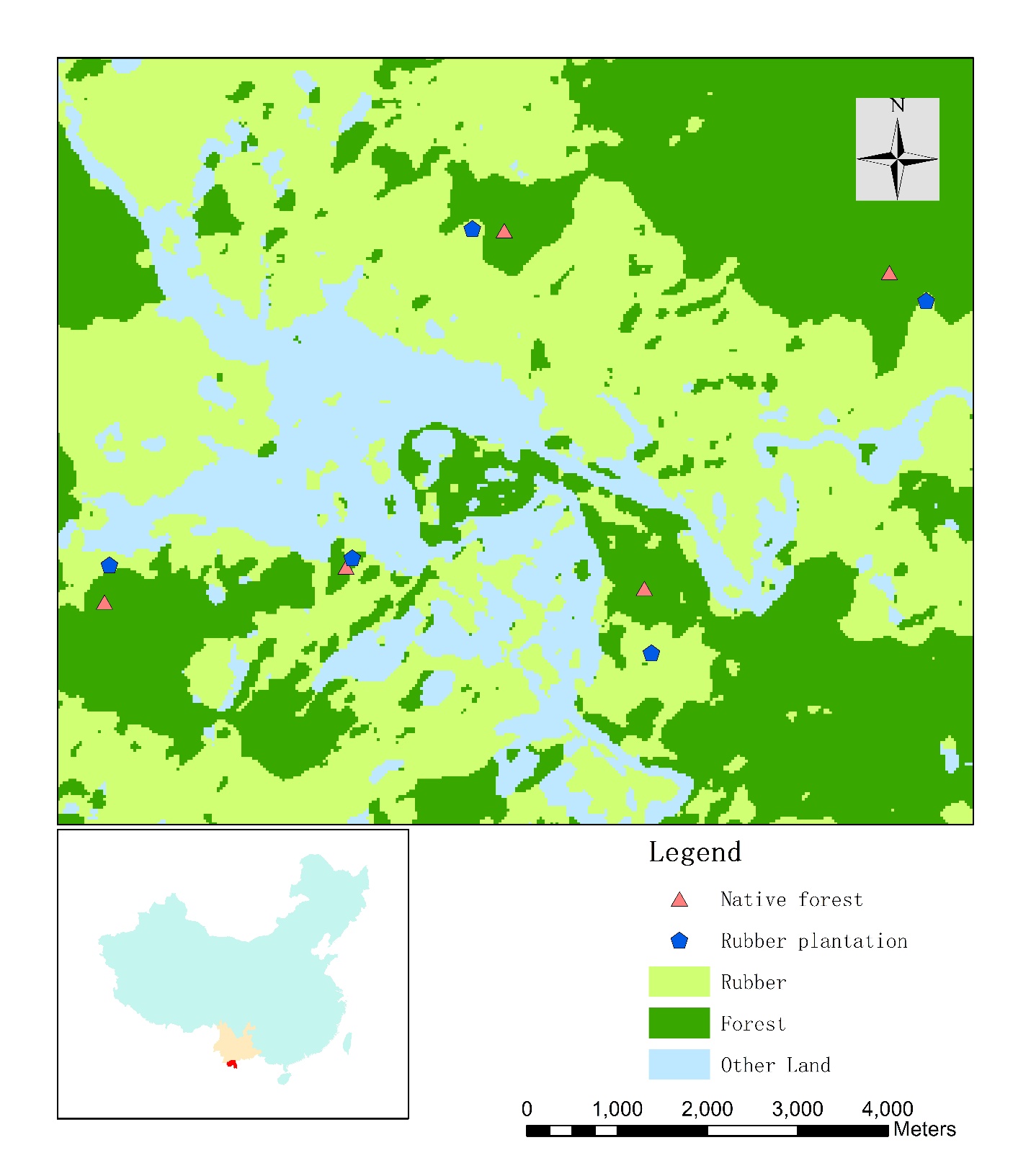


**Figure S4** Map of the study area showing the matched pair sites sampled. This map was developed using ArcGIS 10.3 (<http://desktop.arcgis.com/en/>). Land cover layers for 2016 were downloaded from Geospatial Data Cloud (<http://www.gscloud.cn/>) using LANDSAT 8 OLI_TIRS (<http://www.gscloud.cn/sources/dataset_desc/411?cdataid=263&pdataid=10&datatype=OLI_TIRS>) for Xishuangbanna prefecture, Yunnan province. Land-use types (i.e. Forest, Rubber and Others) were classified using ENVI software version 5.3 (<http://geospatial-solutions.com/envi-3-6-adds-lidar-analysis/>).

Data Cloud is a Support and Service infrastructure hosted by the Chinese Academy of Sciences (<http://www.csdb.cn/>). Geospatial data for China is curated and available to registered users through the Computer Network Information Center (CNIC), an institute of the Chinese Academy of Sciences.


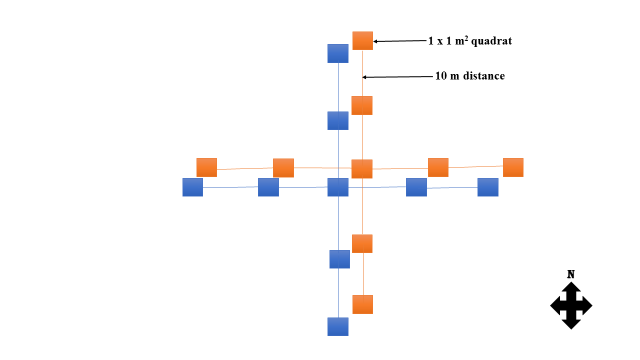


**Figure S5** Schematic representation of sampling design. In each site, nine 1 x 1 m^2^ quadrats (placed 10 m apart; one in the middle and two each in north, east, west and south directions) were established in each land-use type. Quadrats of the preceding month’s sampling were placed within 1-2 m of the current month’s ones. The blue diagram represents current month’s sampling (e.g. January) and the orange diagram represents preceding month’s sampling (e.g. February). This figure was drawn using Microsoft PowerPoint 2016 (<https://products.office.com/en-us/powerpoint>).

**Figure S6**. Relationship among conventional versus rarefied OTU richness estimators

**Figure S7**. Relationship among environmental predictors

**Table S1**. Pairwise contrast compositional differences between months (Jan –Dec) in native forests and rubber plantations. Analysis were performed using permutational multivariate analysis of variance (PerMANOVA) followed by pairwise comparisons between group levels (months) with corrections for multiple testing using the “fdr” method. Significance codes: 0 ‘***’ 0.001 ‘**’ 0.01 ‘*’ 0.05 ‘.’ 0.1 ‘ ’ 1

| **Native forests** | | | | |  | **Rubber plantations** | | | |
| --- | --- | --- | --- | --- | --- | --- | --- | --- | --- |
| Pairs | F.Model | R^2^ | p.value | p.adjusted |  | F.Model | R^2^ | p.value | p.adjusted |
| Jan vs Feb | 1.075 | 0.118 | 0.275 | 0.318 |  | 1.269 | 0.137 | 0.115 | 0.152 |
| Jan vs Mar | 0.919 | 0.103 | 0.651 | 0.671 |  | 1.132 | 0.124 | 0.237 | 0.274 |
| Jan vs Apr | 1.274 | 0.137 | 0.052 | 0.072 |  | 1.676 | 0.173 | 0.010 | 0.027 * |
| Jan vs May | 1.522 | 0.160 | 0.027 | 0.041 * |  | 1.567 | 0.164 | 0.023 | 0.040 * |
| Jan vs Jun | 1.433 | 0.152 | 0.026 | 0.040 * |  | 1.686 | 0.174 | 0.007 | 0.027 * |
| Jan vs Jul | 1.825 | 0.186 | 0.008 | 0.026 * |  | 3.081 | 0.278 | 0.008 | 0.027 * |
| Jan vs Aug | 1.434 | 0.152 | 0.005 | 0.026 * |  | 1.987 | 0.199 | 0.008 | 0.027 * |
| Jan vs Sep | 1.315 | 0.141 | 0.077 | 0.102 |  | 1.908 | 0.193 | 0.009 | 0.027 * |
| Jan vs Oct | 1.155 | 0.126 | 0.138 | 0.175 |  | 1.134 | 0.124 | 0.233 | 0.274 |
| Jan vs Nov | 1.139 | 0.125 | 0.213 | 0.256 |  | 1.131 | 0.124 | 0.131 | 0.170 |
| Jan vs Dec | 1.041 | 0.115 | 0.438 | 0.459 |  | 1.480 | 0.156 | 0.041 | 0.062 |
| Feb vs Mar | 1.048 | 0.116 | 0.350 | 0.379 |  | 0.796 | 0.091 | 0.865 | 0.889 |
| Feb vs Apr | 1.268 | 0.137 | 0.058 | 0.078 |  | 1.246 | 0.135 | 0.078 | 0.107 |
| Feb vs May | 2.027 | 0.202 | 0.008 | 0.026 * |  | 1.454 | 0.154 | 0.023 | 0.040 * |
| Feb vs Jun | 1.840 | 0.187 | 0.010 | 0.026 * |  | 1.288 | 0.139 | 0.204 | 0.249 |
| Feb vs Jul | 2.946 | 0.269 | 0.012 | 0.026 * |  | 2.918 | 0.267 | 0.012 | 0.027 * |
| Feb vs Aug | 2.411 | 0.232 | 0.011 | 0.026 * |  | 1.730 | 0.178 | 0.013 | 0.027 * |
| Feb vs Sep | 2.358 | 0.228 | 0.014 | 0.028 * |  | 1.772 | 0.181 | 0.007 | 0.027 * |
| Feb vs Oct | 2.046 | 0.204 | 0.008 | 0.026 * |  | 1.517 | 0.159 | 0.050 | 0.073 |
| Feb vs Nov | 1.949 | 0.196 | 0.011 | 0.026 * |  | 1.831 | 0.186 | 0.004 | 0.027 * |
| Feb vs Dec | 1.991 | 0.199 | 0.011 | 0.026 * |  | 2.095 | 0.208 | 0.009 | 0.027 * |
| Mar vs Apr | 1.149 | 0.126 | 0.206 | 0.256 |  | 0.983 | 0.109 | 0.444 | 0.488 |
| Mar vs May | 1.863 | 0.189 | 0.011 | 0.026 * |  | 1.364 | 0.146 | 0.039 | 0.061 |
| Mar vs Jun | 1.555 | 0.163 | 0.015 | 0.028 * |  | 1.444 | 0.153 | 0.027 | 0.045 * |
| Mar vs Jul | 2.082 | 0.207 | 0.005 | 0.026 * |  | 2.900 | 0.266 | 0.011 | 0.027 * |
| Mar vs Aug | 1.629 | 0.169 | 0.016 | 0.029 * |  | 1.674 | 0.173 | 0.029 | 0.047 * |
| Mar vs Sep | 1.505 | 0.158 | 0.050 | 0.070 |  | 1.717 | 0.177 | 0.020 | 0.038 * |
| Mar vs Oct | 1.439 | 0.152 | 0.023 | 0.036 * |  | 1.485 | 0.157 | 0.052 | 0.073 |
| Mar vs Nov | 1.590 | 0.166 | 0.011 | 0.026 * |  | 1.734 | 0.178 | 0.005 | 0.027 * |
| Mar vs Dec | 1.763 | 0.181 | 0.007 | 0.026 * |  | 1.869 | 0.189 | 0.008 | 0.027 * |
| Apr vs May | 1.454 | 0.154 | 0.020 | 0.033 * |  | 1.457 | 0.154 | 0.013 | 0.027 * |
| Apr vs Jun | 1.510 | 0.159 | 0.023 | 0.036 * |  | 1.761 | 0.180 | 0.012 | 0.027 * |
| Apr vs Jul | 3.019 | 0.274 | 0.006 | 0.026 * |  | 3.904 | 0.328 | 0.007 | 0.027 * |
| Apr vs Aug | 2.537 | 0.241 | 0.008 | 0.026 * |  | 2.312 | 0.224 | 0.008 | 0.027 * |
| Apr vs Sep | 2.103 | 0.208 | 0.017 | 0.030 * |  | 2.265 | 0.221 | 0.007 | 0.027 * |
| Apr vs Oct | 2.148 | 0.212 | 0.009 | 0.026 * |  | 1.748 | 0.179 | 0.007 | 0.027 * |
| Apr vs Nov | 1.840 | 0.187 | 0.009 | 0.026 * |  | 2.332 | 0.226 | 0.006 | 0.027 * |
| Apr vs Dec | 1.855 | 0.188 | 0.009 | 0.026 * |  | 2.335 | 0.226 | 0.010 | 0.027 * |
| May vs Jun | 1.138 | 0.124 | 0.237 | 0.279 |  | 0.777 | 0.089 | 0.876 | 0.889 |
| May vs Jul | 2.682 | 0.251 | 0.012 | 0.026 * |  | 2.265 | 0.221 | 0.013 | 0.027 * |
| May vs Aug | 2.407 | 0.231 | 0.010 | 0.026 * |  | 1.548 | 0.162 | 0.040 | 0.061 |
| May vs Sep | 2.131 | 0.210 | 0.007 | 0.026 * |  | 1.682 | 0.174 | 0.009 | 0.027 * |
| May vs Oct | 2.264 | 0.221 | 0.014 | 0.028 * |  | 0.943 | 0.105 | 0.509 | 0.551 |
| May vs Nov | 1.777 | 0.182 | 0.012 | 0.026 * |  | 1.541 | 0.162 | 0.010 | 0.027 * |
| May vs Dec | 1.845 | 0.187 | 0.008 | 0.026 * |  | 1.908 | 0.193 | 0.009 | 0.027 * |
| Jun vs Jul | 1.771 | 0.181 | 0.010 | 0.026 * |  | 1.818 | 0.185 | 0.007 | 0.027 * |
| Jun vs Aug | 1.625 | 0.169 | 0.010 | 0.026 * |  | 1.235 | 0.134 | 0.150 | 0.190 |
| Jun vs Sep | 1.643 | 0.170 | 0.012 | 0.026 * |  | 1.562 | 0.163 | 0.023 | 0.040 * |
| Jun vs Oct | 1.784 | 0.182 | 0.015 | 0.028 * |  | 1.127 | 0.124 | 0.250 | 0.280 |
| Jun vs Nov | 1.736 | 0.178 | 0.008 | 0.026 * |  | 1.602 | 0.167 | 0.006 | 0.027 * |
| Jun vs Dec | 2.026 | 0.202 | 0.011 | 0.026 * |  | 1.785 | 0.182 | 0.013 | 0.027 * |
| Jul vs Aug | 1.078 | 0.119 | 0.364 | 0.387 |  | 1.244 | 0.135 | 0.157 | 0.196 |
| Jul vs Sep | 1.280 | 0.138 | 0.113 | 0.146 |  | 1.643 | 0.170 | 0.019 | 0.037 * |
| Jul vs Oct | 1.645 | 0.171 | 0.010 | 0.026 * |  | 2.091 | 0.207 | 0.013 | 0.027 * |
| Jul vs Nov | 2.022 | 0.202 | 0.015 | 0.028 * |  | 2.410 | 0.232 | 0.011 | 0.027 * |
| Jul vs Dec | 2.530 | 0.240 | 0.009 | 0.026 * |  | 2.121 | 0.210 | 0.012 | 0.027 * |
| Aug vs Sep | 0.736 | 0.084 | 0.923 | 0.923 |  | 1.154 | 0.126 | 0.223 | 0.268 |
| Aug vs Oct | 1.071 | 0.118 | 0.295 | 0.336 |  | 1.513 | 0.159 | 0.086 | 0.116 |
| Aug vs Nov | 1.539 | 0.161 | 0.039 | 0.056 |  | 2.004 | 0.200 | 0.014 | 0.028 * |
| Aug vs Dec | 2.166 | 0.213 | 0.013 | 0.028 * |  | 1.844 | 0.187 | 0.007 | 0.027 * |
| Sep vs Oct | 0.813 | 0.092 | 0.875 | 0.888 |  | 1.193 | 0.130 | 0.241 | 0.274 |
| Sep vs Nov | 1.204 | 0.131 | 0.213 | 0.256 |  | 1.498 | 0.158 | 0.051 | 0.073 |
| Sep vs Dec | 1.754 | 0.180 | 0.018 | 0.030 * |  | 1.401 | 0.149 | 0.026 | 0.044 * |
| Oct vs Nov | 1.005 | 0.112 | 0.347 | 0.379 |  | 0.709 | 0.081 | 0.942 | 0.942 |
| Oct vs Dec | 1.388 | 0.148 | 0.038 | 0.056 |  | 0.921 | 0.103 | 0.638 | 0.668 |
| Nov vs Dec | 1.095 | 0.120 | 0.307 | 0.343 |  | 0.964 | 0.107 | 0.560 | 0.596 |

**Table S2**. Pairwise comparison of species richness between months (Jan –Dec) using the Tukey’s Honest Significant Difference (TukeyHSD) test. Significance codes: 0 ‘***’ 0.001 ‘**’ 0.01 ‘*’ 0.05 ‘.’ 0.1 ‘ ’ 1

| **Pairs** | **Difference in means** | **Lower interval** | **Upper interval** | **Adjusted p-value** |
| --- | --- | --- | --- | --- |
| Aug-Apr | 69.4 | -36.379 | 175.179 | 0.522 |
| Dec-Apr | 57.8 | -47.979 | 163.579 | 0.767 |
| Feb-Apr | -32.6 | -138.379 | 73.179 | 0.995 |
| Jan-Apr | 28 | -77.779 | 133.779 | 0.999 |
| Jul-Apr | 80.8 | -24.979 | 186.579 | 0.296 |
| Jun-Apr | 26.4 | -79.379 | 132.179 | 0.999 |
| Mar-Apr | 16 | -89.779 | 121.779 | 1.000 |
| May-Apr | 73.6 | -32.179 | 179.379 | 0.433 |
| Nov-Apr | 73.6 | -32.179 | 179.379 | 0.433 |
| Oct-Apr | 33.4 | -72.379 | 139.179 | 0.994 |
| Sep-Apr | 64.4 | -41.379 | 170.179 | 0.631 |
| Dec-Aug | -11.6 | -117.379 | 94.179 | 1.000 |
| Feb-Aug | -102 | -207.779 | 3.779 | 0.068 |
| Jan-Aug | -41.4 | -147.179 | 64.379 | 0.968 |
| Jul-Aug | 11.4 | -94.379 | 117.179 | 1.000 |
| Jun-Aug | -43 | -148.779 | 62.779 | 0.959 |
| Mar-Aug | -53.4 | -159.179 | 52.379 | 0.844 |
| May-Aug | 4.2 | -101.579 | 109.979 | 1.000 |
| Nov-Aug | 4.2 | -101.579 | 109.979 | 1.000 |
| Oct-Aug | -36 | -141.779 | 69.779 | 0.989 |
| Sep-Aug | -5 | -110.779 | 100.779 | 1.000 |
| Feb-Dec | -90.4 | -196.179 | 15.379 | 0.161 |
| Jan-Dec | -29.8 | -135.579 | 75.979 | 0.998 |
| Jul-Dec | 23 | -82.779 | 128.779 | 1.000 |
| Jun-Dec | -31.4 | -137.179 | 74.379 | 0.996 |
| Mar-Dec | -41.8 | -147.579 | 63.979 | 0.966 |
| May-Dec | 15.8 | -89.979 | 121.579 | 1.000 |
| Nov-Dec | 15.8 | -89.979 | 121.579 | 1.000 |
| Oct-Dec | -24.4 | -130.179 | 81.379 | 1.000 |
| Sep-Dec | 6.6 | -99.179 | 112.379 | 1.000 |
| Jan-Feb | 60.6 | -45.179 | 166.379 | 0.712 |
| Jul-Feb | 113.4 | 7.621 | 219.179 | 0.026 * |
| Jun-Feb | 59 | -46.779 | 164.779 | 0.744 |
| Mar-Feb | 48.6 | -57.179 | 154.379 | 0.909 |
| May-Feb | 106.2 | 0.421 | 211.979 | 0.048 * |
| Nov-Feb | 106.2 | 0.421 | 211.979 | 0.048 * |
| Oct-Feb | 66 | -39.779 | 171.779 | 0.597 |
| Sep-Feb | 97 | -8.779 | 202.779 | 0.100 |
| Jul-Jan | 52.8 | -52.979 | 158.579 | 0.853 |
| Jun-Jan | -1.6 | -107.379 | 104.179 | 1.000 |
| Mar-Jan | -12 | -117.779 | 93.779 | 1.000 |
| May-Jan | 45.6 | -60.179 | 151.379 | 0.939 |
| Nov-Jan | 45.6 | -60.179 | 151.379 | 0.939 |
| Oct-Jan | 5.4 | -100.379 | 111.179 | 1.000 |
| Sep-Jan | 36.4 | -69.379 | 142.179 | 0.988 |
| Jun-Jul | -54.4 | -160.179 | 51.379 | 0.828 |
| Mar-Jul | -64.8 | -170.579 | 40.979 | 0.623 |
| May-Jul | -7.2 | -112.979 | 98.579 | 1.000 |
| Nov-Jul | -7.2 | -112.979 | 98.579 | 1.000 |
| Oct-Jul | -47.4 | -153.179 | 58.379 | 0.922 |
| Sep-Jul | -16.4 | -122.179 | 89.379 | 1.000 |
| Mar-Jun | -10.4 | -116.179 | 95.379 | 1.000 |
| May-Jun | 47.2 | -58.579 | 152.979 | 0.924 |
| Nov-Jun | 47.2 | -58.579 | 152.979 | 0.924 |
| Oct-Jun | 7 | -98.779 | 112.779 | 1.000 |
| Sep-Jun | 38 | -67.779 | 143.779 | 0.983 |
| May-Mar | 57.6 | -48.179 | 163.379 | 0.771 |
| Nov-Mar | 57.6 | -48.179 | 163.379 | 0.771 |
| Oct-Mar | 17.4 | -88.379 | 123.179 | 1.000 |
| Sep-Mar | 48.4 | -57.379 | 154.179 | 0.911 |
| Nov-May | 0 | -105.779 | 105.779 | 1.000 |
| Oct-May | -40.2 | -145.979 | 65.579 | 0.974 |
| Sep-May | -9.2 | -114.979 | 96.579 | 1.000 |
| Oct-Nov | -40.2 | -145.979 | 65.579 | 0.974 |
| Sep-Nov | -9.2 | -114.979 | 96.579 | 1.000 |
| Sep-Oct | 31 | -74.779 | 136.779 | 0.997 |

**Table 3**. Model fit parameters from Canonical Correspondence Analysis (CCA) gradient analysis of arthropod community composition in forests and rubber across arthropod taxa. The significance of the CCA model, CCA axes and environmental variables was tested using 999 permutations and only variables with p < 0.05 were considered to have significant effects. *Canopy openness* was measured from nine hemispherical (true-color fisheye) photographs using the Gap Light Analyzer (GLA) version 2.0 software. *Litter thickness* was measured at three points within each of the nine 1 x 1 m^2^ quadrats (27 measurements per site) with a ruler. *Soil moisture content (SMC)* was measured from 200 g of soil collected from the nine 1 x 1 m^2^ quadrats using a corer (10 cm depth), homogenized, oven-dried to constant weight and reweighed. *Temperature* and *Humidity* were measured at 30-minutes intervals over the whole year using iButton Hydrochron data loggers installed at 0.5 m above the ground. Significance codes: 0 ‘***’ 0.001 ‘**’ 0.01 ‘*’ 0.05 ‘.’ 0.1 ‘ ’ 1

|  | **Native forests** | | | | |  | **Rubber plantations** | | | |
| --- | --- | --- | --- | --- | --- | --- | --- | --- | --- | --- |
| **Taxa** | **CCA model** | **Df** | **ChiSquare** | **F** | **Pr(>F)** |  | **Df** | **ChiSquare** | **F** | **Pr(>F)** |
|  |  |  |  |  |  |  |  |  |  |  |
| Araneae | Model | 5 | 0.84 | 1.07 | 0.227 |  | 5 | 1.43 | 1.50 | 0.003 ** |
|  | Residual | 52 | 8.13 |  |  |  | 47 | 9.00 |  |  |
|  | **Axis** |  |  |  |  |  |  |  |  |  |
|  | CCA1 | 1 | 0.23 | 1.49 | 0.633 |  | 1 | 0.57 | 2.96 | 0.006 ** |
|  | CCA2 | 1 | 0.22 | 1.40 | 0.581 |  | 1 | 0.36 | 1.87 | 0.216 |
|  | CCA3 | 1 | 0.19 | 1.18 | 0.756 |  | 1 | 0.25 | 1.32 | 0.602 |
|  | CCA4 | 1 | 0.12 | 0.74 | 0.989 |  | 1 | 0.14 | 0.72 | 0.969 |
|  | CCA5 | 1 | 0.09 | 0.55 | 0.993 |  | 1 | 0.12 | 0.60 | 0.891 |
|  | Residual | 52 | 8.13 |  |  |  | 47 | 9.00 |  |  |
|  | **Variables** |  |  |  |  |  |  |  |  |  |
|  | Canopy openness | 1 | 0.22 | 1.40 | 0.053 * |  | 1 | 0.32 | 1.68 | 0.052 * |
|  | Litter thickness | 1 | 0.14 | 0.90 | 0.620 |  | 1 | 0.33 | 1.70 | 0.039 * |
|  | Soil moisture | 1 | 0.14 | 0.90 | 0.665 |  | 1 | 0.33 | 1.71 | 0.023 * |
|  | Temperature | 1 | 0.13 | 0.86 | 0.711 |  | 1 | 0.15 | 0.80 | 0.703 |
|  | Humidity | 1 | 0.21 | 1.31 | 0.147 |  | 1 | 0.31 | 1.59 | 0.049 * |
|  | Residual | 52 | 8.13 |  |  |  | 47 | 9.00 |  |  |
|  |  |  |  |  |  |  |  |  |  |  |
| Blattodea | Model | 5 | 0.43 | 0.92 | 0.718 |  | 5 | 0.52 | 1.07 | 0.274 |
|  | Residual | 54 | 5.07 |  |  |  | 54 | 5.30 |  |  |
|  | **Axis** |  |  |  |  |  |  |  |  |  |
|  | CCA1 | 1 | 0.14 | 1.47 | 0.576 |  | 1 | 0.19 | 1.89 | 0.154 |
|  | CCA2 | 1 | 0.11 | 1.15 | 0.870 |  | 1 | 0.12 | 1.23 | 0.758 |
|  | CCA3 | 1 | 0.08 | 0.81 | 0.999 |  | 1 | 0.09 | 0.92 | 0.945 |
|  | CCA4 | 1 | 0.07 | 0.75 | 0.992 |  | 1 | 0.08 | 0.84 | 0.918 |
|  | CCA5 | 1 | 0.04 | 0.43 | 1.000 |  | 1 | 0.05 | 0.46 | 0.995 |
|  | Residual | 54 | 5.07 |  |  |  | 54 | 5.30 |  |  |
|  | **Variables** |  |  |  |  |  |  |  |  |  |
|  | Canopy openness | 1 | 0.07 | 0.72 | 0.945 |  | 1 | 0.07 | 0.74 | 0.759 |
|  | Litter thickness | 1 | 0.09 | 0.92 | 0.592 |  | 1 | 0.10 | 1.01 | 0.404 |
|  | Soil moisture | 1 | 0.09 | 0.95 | 0.513 |  | 1 | 0.10 | 1.06 | 0.377 |
|  | Temperature | 1 | 0.09 | 0.99 | 0.446 |  | 1 | 0.15 | 1.57 | 0.012 * |
|  | Humidity | 1 | 0.10 | 1.04 | 0.355 |  | 1 | 0.09 | 0.96 | 0.496 |
|  | Residual | 54 | 5.07 |  |  |  | 54 | 5.30 |  |  |
|  |  |  |  |  |  |  |  |  |  |  |
| Coleoptera | Model | 5 | 0.95 | 1.13 | 0.002 ** |  | 5 | 1.22 | 1.28 | 0.001 *** |
|  | Residual | 54 | 9.07 |  |  |  | 54 | 10.29 |  |  |
|  | **Axis** |  |  |  |  |  |  |  |  |  |
|  | CCA1 | 1 | 0.24 | 1.42 | 0.025 * |  | 1 | 0.34 | 1.78 | 0.009 ** |
|  | CCA2 | 1 | 0.22 | 1.30 | 0.139 |  | 1 | 0.27 | 1.42 | 0.101 |
|  | CCA3 | 1 | 0.19 | 1.13 | 0.564 |  | 1 | 0.24 | 1.24 | 0.306 |
|  | CCA4 | 1 | 0.17 | 0.99 | 0.853 |  | 1 | 0.21 | 1.10 | 0.471 |
|  | CCA5 | 1 | 0.14 | 0.83 | 0.919 |  | 1 | 0.16 | 0.86 | 0.881 |
|  | Residual | 54 | 9.07 |  |  |  | 54 | 10.29 |  |  |
|  | **Variables** |  |  |  |  |  |  |  |  |  |
|  | Canopy openness | 1 | 0.17 | 1.01 | 0.480 |  | 1 | 0.25 | 1.29 | 0.019 * |
|  | Litter thickness | 1 | 0.18 | 1.01 | 0.281 |  | 1 | 0.29 | 1.54 | 0.005 ** |
|  | Soil moisture | 1 | 0.19 | 1.10 | 0.128 |  | 1 | 0.21 | 1.11 | 0.171 |
|  | Temperature | 1 | 0.20 | 1.20 | 0.024 * |  | 1 | 0.22 | 1.17 | 0.080 . |
|  | Humidity | 1 | 0.22 | 1.30 | 0.002 ** |  | 1 | 0.25 | 1.28 | 0.018 * |
|  | Residual | 54 | 9.07 |  |  |  | 54 | 10.29 |  |  |
|  |  |  |  |  |  |  |  |  |  |  |
| Diptera | Model | 5 | 1.12 | 1.08 | 0.071 . |  | 5 | 1.53 | 1.01 | 0.382 |
|  | Residual | 54 | 11.27 |  |  |  | 54 | 16.34 |  |  |
|  | **Axis** |  |  |  |  |  |  |  |  |  |
|  | CCA1 | 1 | 0.29 | 1.38 | 0.066 . |  | 1 | 0.44 | 1.46 | 0.039 * |
|  | CCA2 | 1 | 0.25 | 1.21 | 0.430 |  | 1 | 0.32 | 1.04 | 0.965 |
|  | CCA3 | 1 | 0.21 | 1.00 | 0.975 |  | 1 | 0.30 | 1.01 | 0.935 |
|  | CCA4 | 1 | 0.20 | 0.95 | 0.939 |  | 1 | 0.27 | 0.89 | 0.945 |
|  | CCA5 | 1 | 0.18 | 0.84 | 0.941 |  | 1 | 0.20 | 0.67 | 0.990 |
|  | Residual | 54 | 11.27 |  |  |  | 54 | 16.34 |  |  |
|  | **Variables** |  |  |  |  |  |  |  |  |  |
|  | Canopy openness | 1 | 0.20 | 0.94 | 0.733 |  | 1 | 0.26 | 0.85 | 0.855 |
|  | Litter thickness | 1 | 0.23 | 1.11 | 0.134 |  | 1 | 0.35 | 1.16 | 0.079 . |
|  | Soil moisture | 1 | 0.21 | 1.00 | 0.483 |  | 1 | 0.31 | 1.02 | 0.426 |
|  | Temperature | 1 | 0.24 | 1.16 | 0.061 . |  | 1 | 0.30 | 1.00 | 0.495 |
|  | Humidity | 1 | 0.24 | 1.17 | 0.062 . |  | 1 | 0.31 | 1.04 | 0.342 |
|  | Residual | 54 | 11.27 |  |  |  | 54 | 16.34 |  |  |
|  |  |  |  |  |  |  |  |  |  |  |
| Hemiptera | Model | 5 | 1.65 | 1.09 | 0.065 . |  | 5 | 1.97 | 1.48 | 0.001 *** |
|  | Residual | 54 | 16.39 |  |  |  | 53 | 14.13 |  |  |
|  | **Axis** |  |  |  |  |  |  |  |  |  |
|  | CCA1 | 1 | 0.40 | 1.32 | 0.308 |  | 1 | 0.58 | 2.19 | 0.004 ** |
|  | CCA2 | 1 | 0.39 | 1.27 | 0.329 |  | 1 | 0.53 | 2.00 | 0.008 ** |
|  | CCA3 | 1 | 0.32 | 1.05 | 0.872 |  | 1 | 0.41 | 1.54 | 0.125 |
|  | CCA4 | 1 | 0.30 | 1.00 | 0.828 |  | 1 | 0.27 | 1.01 | 0.778 |
|  | CCA5 | 1 | 024 | 0.79 | 0.948 |  | 1 | 0.18 | 0.67 | 0.972 |
|  | Residual | 54 | 16.39 |  |  |  | 53 | 14.13 |  |  |
|  | **Variables** |  |  |  |  |  |  |  |  |  |
|  | Canopy openness | 1 | 0.30 | 0.98 | 0.525 |  | 1 | 0.47 | 1.76 | 0.003 ** |
|  | Litter thickness | 1 | 0.36 | 1.17 | 0.089 . |  | 1 | 0.38 | 1.44 | 0.067 . |
|  | Soil moisture | 1 | 0.31 | 1.04 | 0.392 |  | 1 | 0.48 | 1.79 | 0.004 ** |
|  | Temperature | 1 | 0.37 | 1.22 | 0.048 * |  | 1 | 0.25 | 0.93 | 0.591 |
|  | Humidity | 1 | 0.31 | 1.03 | 0.395 |  | 1 | 0.40 | 1.49 | 0.008 ** |
|  | Residual | 54 | 16.39 |  |  |  | 53 | 14.13 |  |  |
|  |  |  |  |  |  |  |  |  |  |  |
| Hymenoptera | Model | 5 | 0.85 | 1.29 | 0.001 *** |  | 5 | 0.85 | 1.35 | 0.001 *** |
|  | Residual | 54 | 7.15 |  |  |  | 54 | 6.76 |  |  |
|  | **Axis** |  |  |  |  |  |  |  |  |  |
|  | CCA1 | 1 | 0.21 | 1.58 | 0.007 ** |  | 1 | 0.27 | 2.15 | 0.001 *** |
|  | CCA2 | 1 | 0.19 | 1.42 | 0.059 . |  | 1 | 0.18 | 1.44 | 0.215 |
|  | CCA3 | 1 | 0.18 | 1.33 | 0.078 . |  | 1 | 0.17 | 1.32 | 0.251 |
|  | CCA4 | 1 | 016 | 1.20 | 0.187 |  | 1 | 0.13 | 1.04 | 0.709 |
|  | CCA5 | 1 | 0.12 | 0.92 | 0.693 |  | 1 | 0.10 | 0.78 | 0.912 |
|  | Residual | 54 | 7.15 |  |  |  | 54 | 6.76 |  |  |
|  | **Variables** |  |  |  |  |  |  |  |  |  |
|  | Canopy openness | 1 | 0.15 | 1.14 | 0.107 |  | 1 | 0.16 | 1.28 | 0.067 . |
|  | Litter thickness | 1 | 0.18 | 1.37 | 0.001 *** |  | 1 | 0.21 | 1.66 | 0.001 *** |
|  | Soil moisture | 1 | 0.19 | 1.44 | 0.001 *** |  | 1 | 0.14 | 1.11 | 0.228 |
|  | Temperature | 1 | 0.15 | 1.10 | 0.190 |  | 1 | 0.15 | 1.22 | 0.085 . |
|  | Humidity | 1 | 0.19 | 1.41 | 0.003 ** |  | 1 | 0.18 | 1.47 | 0.007 ** |
|  | Residual | 54 | 7.15 |  |  |  | 54 | 6.76 |  |  |
|  |  |  |  |  |  |  |  |  |  |  |
| Isoptera | Model | 5 | 0.87 | 1.35 | 0.002 ** |  | 5 | 0.81 | 1.55 | 0.001 *** |
|  | Residual | 54 | 6.99 |  |  |  | 51 | 5.34 |  |  |
|  | **Axis** |  |  |  |  |  |  |  |  |  |
|  | CCA1 | 1 | 0.27 | 2.05 | 0.022 * |  | 1 | 0.29 | 2.77 | 0.004 ** |
|  | CCA2 | 1 | 0.21 | 1.61 | 0.198 |  | 1 | 0.18 | 1.71 | 0.195 |
|  | CCA3 | 1 | 0.16 | 1.20 | 0.700 |  | 1 | 0.14 | 1.36 | 0.434 |
|  | CCA4 | 1 | 0.13 | 1.02 | 0.779 |  | 1 | 0.12 | 1.14 | 0.563 |
|  | CCA5 | 1 | 0.11 | 0.85 | 0.730 |  | 1 | 0.08 | 0.76 | 0.842 |
|  | Residual | 54 | 6.99 |  |  |  | 51 | 5.34 |  |  |
|  | **Variables** |  |  |  |  |  |  |  |  |  |
|  | Canopy openness | 1 | 0.17 | 1.33 | 0.066 . |  | 1 | 0.21 | 1.99 | 0.002 ** |
|  | Litter thickness | 1 | 0.16 | 1.22 | 0.189 |  | 1 | 0.16 | 1.49 | 0.085 . |
|  | Soil moisture | 1 | 0.23 | 1.78 | 0.004 ** |  | 1 | 0.20 | 1.92 | 0.001 *** |
|  | Temperature | 1 | 0.17 | 1.27 | 0.144 |  | 1 | 0.13 | 1.24 | 0.182 |
|  | Humidity | 1 | 0.15 | 1.13 | 0.244 |  | 1 | 0.12 | 1.09 | 0.307 |
|  | Residual | 54 | 6.99 |  |  |  | 51 | 5.34 |  |  |
|  |  |  |  |  |  |  |  |  |  |  |
| Orthoptera | Model | 5 | 1.65 | 1.36 | 0.004 ** |  | 5 | 1.52 | 1.23 | 0.106 |
|  | Residual | 52 | 12.63 |  |  |  | 49 | 12.10 |  |  |
|  | **Axis** |  |  |  |  |  |  |  |  |  |
|  | CCA1 | 1 | 0.59 | 2.43 | 0.004 ** |  | 1 | 0.58 | 2.34 | 0.072 . |
|  | CCA2 | 1 | 0.46 | 1.88 | 0.012 * |  | 1 | 0.31 | 1.27 | 0.731 |
|  | CCA3 | 1 | 0.25 | 1.04 | 0.895 |  | 1 | 0.27 | 1.11 | 0.761 |
|  | CCA4 | 1 | 0.19 | 0.77 | 0.984 |  | 1 | 0.21 | 0.85 | 0.892 |
|  | CCA5 | 1 | 0.17 | 0.68 | 0.934 |  | 1 | 0.14 | 0.58 | 0.963 |
|  | Residual | 52 | 12.63 |  |  |  | 49 | 12.10 |  |  |
|  | **Variables** |  |  |  |  |  |  |  |  |  |
|  | Canopy openness | 1 | 0.44 | 1.80 | 0.011 * |  | 1 | 0.35 | 1.40 | 0.191 |
|  | Litter thickness | 1 | 0.21 | 0.86 | 0.642 |  | 1 | 0.22 | 0.87 | 0.546 |
|  | Soil moisture | 1 | 0.43 | 1.79 | 0.001 *** |  | 1 | 0.38 | 1.54 | 0.034 * |
|  | Temperature | 1 | 0.26 | 1.06 | 0.300 |  | 1 | 0.28 | 1.11 | 0.299 |
|  | Humidity | 1 | 0.31 | 1.27 | 0.065 . |  | 1 | 0.30 | 1.21 | 0.218 |
|  | Residual | 52 | 12.63 |  |  |  | 49 | 12.10 |  |  |

**Table S4**. Factors that influence arthropod species richness in forests and in rubber computed using generalized linear mixed-effects regression with random effects for site. Model <- glmer (Richness ~ Canopy openness + Litter thickness + Soil moisture content + Temperature + Humidity + (1|Plot), method = "REML"). Significance codes: 0 ‘***’ 0.001 ‘**’ 0.01 ‘*’ 0.05 ‘.’ 0.1 ‘ ’ 1

| **Taxa** |  | **Native forests** | | | | **Rubber plantations** | | | | |
| --- | --- | --- | --- | --- | --- | --- | --- | --- | --- | --- |
| Araneae | Random effects: |  |  |  |  |  |  |  |  |  |
|  | Groups | Variance | Std.Dev. |  |  |  | Variance | Std.Dev. |  |  |
|  | Site (n = 5) | 0.05 | 0.21 |  |  |  | 0.00 | 0.00 |  |  |
|  | Residuals | 0.26 | 0.51 |  |  |  | 0.43 | 0.66 |  |  |
|  | Fixed effects: |  |  |  |  |  |  |  |  |  |
|  |  | Estimate | Std.Error | t value | p-value |  | Estimate | Std.Error | z value | p-value |
|  | (Intercept) | 2.01 | 0.12 | 17.45 |  |  | 1.36 | 0.08 | 16.18 |  |
|  | Canopy openness | 0.08 | 0.08 | 0.95 | 0.344 |  | 0.17 | 0.15 | 1.13 | 0.256 |
|  | Litter thickness | -0.15 | 0.10 | -1.44 | 0.150 |  | -0.25 | 0.13 | -1.84 | 0.064 |
|  | Soil moisture | 0.23 | 0.10 | 2.31 | 0.021 * |  | 0.13 | 0.11 | 1.17 | 0.239 |
|  | Temperature | -0.11 | 0.08 | -1.38 | 0.167 |  | 0.01 | 0.14 | 0.13 | 0.891 |
|  | Humidity | 0.02 | 0.11 | 0.18 | 0.855 |  | -0.12 | 0.15 | -0.79 | 0.425 |
|  |  |  |  |  |  |  |  |  |  |  |
| Blattodea | Random effects: |  |  |  |  |  |  |  |  |  |
|  | Groups | Variance | Std.Dev. |  |  |  | Variance | Std.Dev. |  |  |
|  | Site (n = 5) | 0.16 | 0.41 |  |  |  | 0.23 | 0.48 |  |  |
|  | Residuals | 1.30 | 1.14 |  |  |  | 1.05 | 1.02 |  |  |
|  | Fixed effects: |  |  |  |  |  |  |  |  |  |
|  |  | Estimate | Std.Error | t value | p-value |  | Estimate | Std.Error | z value | p-value |
|  | (Intercept) | 5.10 | 0.23 | 21.63 |  |  | 3.76 | 0.25 | 14.89 |  |
|  | Canopy openness | 0.07 | 0.18 | 0.42 | 0.669 |  | -0.14 | 0.24 | -0.60 | 0.548 |
|  | Litter thickness | -0.65 | 0.23 | -2.81 | 0.004 ** |  | -0.01 | 0.22 | -0.04 | 0.964 |
|  | Soil moisture | 0.20 | 0.22 | 0.91 | 0.359 |  | 0.20 | 0.24 | 0.82 | 0.407 |
|  | Temperature | 0.23 | 0.18 | 1.31 | 0.190 |  | -0.20 | 0.24 | -0.82 | 0.411 |
|  | Humidity | 0.44 | 0.25 | 1.71 | 0.086 |  | 0.56 | 0.25 | 2.21 | 0.026 * |
|  |  |  |  |  |  |  |  |  |  |  |
| Coleoptera | Random effects: |  |  |  |  |  |  |  |  |  |
|  | Groups | Variance | Std.Dev. |  |  |  | Variance | Std.Dev. |  |  |
|  | Site (n = 5) | 0.00 | 0.00 |  |  |  | 0.02 | 0.17 |  |  |
|  | Residuals | 0.74 | 0.86 |  |  |  | 0.92 | 0.96 |  |  |
|  | Fixed effects: |  |  |  |  |  |  |  |  |  |
|  |  | Estimate | Std.Error | t value | p-value |  | Estimate | Std.Error | z value | p-value |
|  | (Intercept) | 5.70 | 0.11 | 51.36 |  |  | 4.57 | 0.14 | 31.40 |  |
|  | Canopy openness | 0.42 | 0.11 | 3.56 | 0.000 *** |  | -0.05 | 0.22 | -0.26 | 0.792 |
|  | Litter thickness | 0.17 | 0.17 | 1.05 | 0.294 |  | -0.17 | 0.20 | -0.84 | 0.395 |
|  | Soil moisture | -0.10 | 0.14 | -0.69 | 0.488 |  | -0.21 | 0.17 | -1.17 | 0.239 |
|  | Temperature | 0.32 | 0.13 | 2.50 | 0.012 * |  | 0.23 | 0.21 | 1.06 | 0.287 |
|  | Humidity | 0.40 | 0.19 | 2.16 | 0.031 * |  | -0.16 | 0.22 | -0.73 | 0.460 |
|  |  |  |  |  |  |  |  |  |  |  |
|  |  |  |  |  |  |  |  |  |  |  |
| Diptera | Random effects: |  |  |  |  |  |  |  |  |  |
|  | Groups | Variance | Std.Dev. |  |  |  | Variance | Std.Dev. |  |  |
|  | Site (n = 5) | 0.05 | 0.23 |  |  |  | 0.07 | 0.26 |  |  |
|  | Residuals | 0.53 | 0.73 |  |  |  | 0.60 | 0.77 |  |  |
|  | Fixed effects: |  |  |  |  |  |  |  |  |  |
|  |  | Estimate | Std.Error | t value | p-value |  | Estimate | Std.Error | z value | p-value |
|  | (Intercept) | 4.85 | 0.13 | 34.77 |  |  | 3.49 | 0.15 | 22.54 |  |
|  | Canopy openness | 0.05 | 0.11 | 0.45 | 0.651 |  | 0.17 | 0.18 | 0.93 | 0.348 |
|  | Litter thickness | 0.00 | 0.14 | 0.01 | 0.994 |  | -0.06 | 0.16 | -0.39 | 0.693 |
|  | Soil moisture | 0.01 | 0.13 | 0.09 | 0.930 |  | 0.05 | 0.16 | 0.31 | 0.754 |
|  | Temperature | 0.15 | 0.11 | 1.31 | 0.191 |  | 0.12 | 0.18 | 0.65 | 0.510 |
|  | Humidity | 0.48 | 0.16 | 2.93 | 0.003 ** |  | -0.01 | 0.18 | -0.03 | 0.975 |
|  |  |  |  |  |  |  |  |  |  |  |
| Hemiptera | Random effects: |  |  |  |  |  |  |  |  |  |
|  | Groups | Variance | Std.Dev. |  |  |  | Variance | Std.Dev. |  |  |
|  | Site (n = 5) | 0.00 | 0.05 |  |  |  | 0.07 | 0.27 |  |  |
|  | Residuals | 0.61 | 0.78 |  |  |  | 0.52 | 0.72 |  |  |
|  | Fixed effects: |  |  |  |  |  |  |  |  |  |
|  |  | Estimate | Std.Error | t value | p-value |  | Estimate | Std.Error | z value | p-value |
|  | (Intercept) | 2.89 | 0.10 | 27.83 |  |  | 2.04 | 0.15 | 13.31 |  |
|  | Canopy openness | 0.09 | 0.11 | 0.84 | 0.397 |  | -0.10 | 0.17 | -0.63 | 0.525 |
|  | Litter thickness | 0.07 | 0.15 | 0.45 | 0.647 |  | 0.14 | 0.15 | 0.88 | 0.375 |
|  | Soil moisture | 0.12 | 0.13 | 0.89 | 0.368 |  | 0.03 | 0.16 | 0.18 | 0.850 |
|  | Temperature | -0.02 | 0.11 | -0.19 | 0.845 |  | -0.02 | 0.17 | -0.14 | 0.883 |
|  | Humidity | 0.07 | 0.17 | 0.43 | 0.666 |  | -0.13 | 0.17 | -0.76 | 0.442 |
|  |  |  |  |  |  |  |  |  |  |  |
| Hymenoptera | Random effects: |  |  |  |  |  |  |  |  |  |
|  | Groups | Variance | Std.Dev. |  |  |  | Variance | Std.Dev. |  |  |
|  | Site (n = 5) | 0.18 | 0.42 |  |  |  | 0.00 | 0.00 |  |  |
|  | Residuals | 0.39 | 0.62 |  |  |  | 0.61 | 0.78 |  |  |
|  | Fixed effects: |  |  |  |  |  |  |  |  |  |
|  |  | Estimate | Std.Error | t value | p-value |  | Estimate | Std.Error | z value | p-value |
|  | (Intercept) | 4.23 | 0.20 | 20.45 |  |  | 3.95 | 0.10 | 39.07 |  |
|  | Canopy openness | 0.11 | 0.10 | 1.02 | 0.305 |  | 0.09 | 0.18 | 0.54 | 0.586 |
|  | Litter thickness | -0.09 | 0.12 | -0.73 | 0.460 |  | -0.13 | 0.16 | -0.82 | 0.414 |
|  | Soil moisture | 0.42 | 0.12 | 3.34 | 0.000 *** |  | 0.10 | 0.13 | 0.78 | 0.437 |
|  | Temperature | 0.31 | 0.10 | 3.10 | 0.001 ** |  | 0.56 | 0.17 | 3.26 | 0.001 ** |
|  | Humidity | 0.07 | 0.14 | 0.52 | 0.597 |  | 0.06 | 0.18 | 0.35 | 0.729 |
|  |  |  |  |  |  |  |  |  |  |  |
| Isoptera | Random effects: |  |  |  |  |  |  |  |  |  |
|  | Groups | Variance | Std.Dev. |  |  |  | Variance | Std.Dev. |  |  |
|  | Site (n = 5) | 0.19 | 0.44 |  |  |  | 0.00 | 0.00 |  |  |
|  | Residuals | 0.70 | 0.83 |  |  |  | 1.27 | 1.13 |  |  |
|  | Fixed effects: |  |  |  |  |  |  |  |  |  |
|  |  | Estimate | Std.Error | t value | p-value |  | Estimate | Std.Error | z value | p-value |
|  | (Intercept) | 2.53 | 0.22 | 11.24 |  |  | 2.59 | 0.14 | 17.75 |  |
|  | Canopy openness | 0.09 | 0.14 | 0.69 | 0.485 |  | 0.07 | 0.26 | 0.30 | 0.761 |
|  | Litter thickness | -0.01 | 0.17 | -0.03 | 0.971 |  | -0.09 | 0.23 | -0.39 | 0.691 |
|  | Soil moisture | -0.48 | 0.16 | -2.89 | 0.003 ** |  | -0.01 | 0.19 | -0.03 | 0.973 |
|  | Temperature | 0.25 | 0.13 | 1.85 | 0.064 |  | -0.06 | 0.25 | -0.26 | 0.790 |
|  | Humidity | 0.42 | 0.19 | 2.23 | 0.025 * |  | 0.39 | 0.26 | 1.47 | 0.139 |
|  |  |  |  |  |  |  |  |  |  |  |
| Orthoptera | Random effects: |  |  |  |  |  |  |  |  |  |
|  | Groups | Variance | Std.Dev. |  |  |  | Variance | Std.Dev. |  |  |
|  | Site (n = 5) | 0.30 | 0.55 |  |  |  | 0.34 | 0.58 |  |  |
|  | Residuals | 1.48 | 1.21 |  |  |  | 1.75 | 1.32 |  |  |
|  | Fixed effects: |  |  |  |  |  |  |  |  |  |
|  |  | Estimate | Std.Error | t value | p-value |  | Estimate | Std.Error | z value | p-value |
|  | (Intercept) | 2.31 | 0.29 | 7.89 |  |  | 2.47 | 0.31 | 7.92 |  |
|  | Canopy openness | 0.26 | 0.20 | 1.32 | 0.185 |  | 0.40 | 0.31 | 1.27 | 0.202 |
|  | Litter thickness | -0.06 | 0.24 | -0.26 | 0.789 |  | -0.10 | 0.29 | -0.34 | 0.728 |
|  | Soil moisture | -0.31 | 0.24 | -1.31 | 0.188 |  | -0.02 | 0.30 | -0.09 | 0.927 |
|  | Temperature | -0.34 | 0.19 | -1.76 | 0.077 |  | -0.21 | 0.31 | -0.66 | 0.505 |
|  | Humidity | 0.27 | 0.27 | 1.00 | 0.316 |  | 0.37 | 0.32 | 1.15 | 0.248 |

**Table S5**. Site characteristics of sampled locations

| **Land-use** | **Code** | **Latitude** | **Longitude** | **Distance to pair (km)** | **Elevation (m)** | **Slope (º)** |
| --- | --- | --- | --- | --- | --- | --- |
| Native forest | F27 | 21.95308 | 101.2571 | 0.35 | 715 | 19 |
| Rubber plantation | R27 | 21.95331 | 101.2536 | 0.35 | 661 | 29 |
| Native forest | F49 | 21.91959 | 101.2396 | 0.12 | 727 | 28 |
| Rubber plantation | R49 | 21.92051 | 101.2403 | 0.12 | 699 | 27 |
| Native forest | F21 | 21.94829 | 101.2983 | 0.51 | 729 | 32 |
| Rubber plantation | R21 | 21.94543 | 101.3022 | 0.51 | 594 | 13 |
| Native forest | F05 | 21.91645 | 101.2136 | 0.42 | 653 | 28 |
| Rubber plantation | R05 | 21.92016 | 101.2142 | 0.42 | 626 | 15 |
| Native forest | F02 | 21.91703 | 101.2715 | 0.72 | 601 | 32 |
| Rubber plantation | R02 | 21.91056 | 101.2722 | 0.72 | 582 | 21 |
